# EEG Evaluation of Stress Exposure on Healthcare Workers During COVID-19 Emergency: Not Just an Impression

**DOI:** 10.3389/fnsys.2022.923576

**Published:** 2022-07-18

**Authors:** Antonella LoMauro, Maria Takeko Molisso, Francesca Mameli, Fabiana Ruggiero, Roberta Ferrucci, Chiara Dellarosa, Giada Aglieco, Andrea Aliverti, Sergio Barbieri, Maurizio Vergari

**Affiliations:** ^1^Dipartimento di Elettronica, Informazione e Bioingegneria. Politecnico di Milano, Milan, Italy; ^2^Unità di Neurofisiopatologia, Fondazione IRCCS Ca' Granda Ospedale Maggiore Policlinico, Milan, Italy; ^3^‘Aldo Ravelli Center', Dipartimento di Scienze della Salute, Università degli Studi di Milano, Milan, Italy; ^4^ASST Santi Paolo e Carlo, III Clinica Neurologica Polo Universitario San Paolo, Milan, Italy; ^5^Dipartimento di Psicologia, Università degli Studi di Milano-Bicocca, Milan, Italy

**Keywords:** COVID-19, stress exposure, EEG, healthcare workers, mental fatigue

## Abstract

Psychological distress among healthcare professionals, although already a common condition, was exacerbated by the COVID-19 pandemic. This effect has been generally self-reported or assessed through questionnaires. We aimed to identify potential abnormalities in the electrical activity of the brain of healthcare workers, operating in different roles during the pandemic. Cortical activity, cognitive performances, sleep, and burnout were evaluated two times in 20 COVID-19 frontline operators (FLCO, median age 29.5 years) and 20 operators who worked in COVID-19-free units (CFO, median 32 years): immediately after the outbreak of the pandemic (first session) and almost 6 months later (second session). FLCO showed higher theta relative power over the entire scalp (FLCO = 19.4%; CFO = 13.9%; *p* = 0.04) and lower peak alpha frequency of electrodes F7 (FLCO = 10.4 Hz; CFO = 10.87 Hz; *p* = 0.017) and F8 (FLCO = 10.47 Hz; CFO = 10.87 Hz; *p* = 0.017) in the first session. FLCO parietal interhemispheric coherence of theta (FLCO I = 0.607; FLCO II = 0.478; *p* = 0.025) and alpha (FLCO I = 0.578; FLCO II = 0.478; *p* = 0.007) rhythms decreased over time. FLCO also showed lower scores in the global cognitive assessment test (FLCO = 22.72 points; CFO = 25.56; *p* = 0.006) during the first session. The quantitative evaluation of the cortical activity might therefore reveal early signs of changes secondary to stress exposure in healthcare professionals, suggesting the implementation of measures to prevent serious social and professional consequences.

## Introduction

Stress among healthcare professionals working in hospitals and medical environments is a common feature. Healthcare professionals usually work long shifts, often overnight, subsequently suffering from sleep deprivation (Ganesan et al., 2019; Di Muzio et al., 2020). In addition, they deal with responsibility and emergencies, performing difficult procedures and treating critically ill patients. If healthcare professionals are not provided with suitable emotional support, the resulting heavy emotional load may therefore lead to severe consequences. Immediate interventions are essential to enhance psychological resilience and improve the healthcare systems' capacity (Pappa et al., 2020). Previous studies established that healthcare workers, especially those assigned to Intensive Care Units, are commonly affected by anxiety, post-traumatic stress syndrome (PTSD), and stress-related disorders, such as fatigue, burnout, and lack of motivation and accomplishment (Mealer et al., 2007; Golubic et al., 2009). This may affect the cognitive performance of clinicians: considering that memory and concentration are essential for healthcare professions, as they impact the decision-making process, the quality of care provided to patients may worsen and, in extreme cases, even personal lives can be jeopardized (Leblanc, 2009).

Although mental stress has been a common issue worldwide for decades, the COVID-19 pandemic has led to a widespread mental crisis whose social and health implications could last for many years ahead (Imperatori et al., 2014; Shevlin et al., 2020). Due to their frontline work with COVID-19 patients, healthcare workers are today even more exposed to the risk of developing physical and mental health issues (Babore et al., 2020; Melnyk et al., 2020; Alonso et al., 2021; Gilleen et al., 2021; Ranieri et al., 2021; Schneider et al., 2021; Testoni et al., 2021; Tauro et al., 2022). Since 2020, many healthcare workers have been facing longer work shifts and a heavier emotional load, dealing with critically ill patients and deaths in a climate of deep uncertainty, living with the fear of being infected and of infecting people close to them, also because of a general lack of adequate personal protective equipment (Ehrlich et al., 2020). Results on the impact of the first wave of the COVID-19 pandemic on the mental health of very large cohorts of healthcare workers have already been published. Alonso et al. administered a cross-sectional, web-based survey to more than 9,000 Spanish healthcare workers, finding that 45.7% of the subjects suffered from mental health conditions such as anxiety, panic attacks, and substance abuse, while 14.5% of them suffered from a disabling mental disorder (Alonso et al., 2021). Gilleen et al. also based their research on an online survey, reporting that nearly a third of the 2,000 British respondents suffered from moderate to severe levels of anxiety and depression (Gilleen et al., 2021). It is therefore clear that the psychological wellbeing of healthcare workers was negatively affected by the pandemic, and this might prove to be a serious problem in the future: therefore, it is crucial for this category of workers to address their mental health issues. However, the existing literature on healthcare work during the COVID-19 pandemic is mostly based on self-reporting through questionnaires, surveys, and interviews, while no study was conducted to assess the presence of potential abnormalities in the brain waves or in the electrical activity of the brain in healthcare workers: it is still undetermined if and how those issues are reflected in the cortical activity in this specific category of operators. The idea behind the present study came after an association between high levels of cumulative life stress and aberrant resting-state EEG was recently found in different categories of patients (Marshall and Cooper, 2017; Ehrhardt et al., 2021; Berretz et al., 2022). Moreover, studies recording EEG signals for mental stress evaluation found stress-related altered rhythms by extracting quantitative parameters from the signals (Seo and Lee, 2010), while spectral analysis of EEG showed decreased alpha power, mainly in the posterior region, and increased theta power in the central region and beta power in the frontal region (Begić et al., 2001). Additionally, it was found that, with increased levels of stress, the frontal connectivity in beta frequencies decreased, while theta and alpha connectivities increased in the parietal region (Imperatori et al., 2014; Lee et al., 2014). Functional connectivity is, therefore, an interesting and useful indicator for the quantification of the interactions between different neuronal networks, as it evaluates the temporal correlation between two or more spatially distant regions (Fingelkurts et al., 2005). Finally, during the COVID-19 pandemic, sleep disturbances were also reported by frontline health care workers, mainly insomnia and sleep disruptions (Stewart et al., 2021).

For these reasons, we conducted a pilot study to ascertain if—and how—recording brain activity might help in objectively identifying the consequences of severe stress exposure. We expect the COVID-19 pandemic to be such a heavy factor of stress in healthcare professionals to negatively affect not only the perceived sleep quality and burnout on working environment but also the cognitive performances, especially memory skills, and to impact on brain activity. In particular, we expect to detect increased theta power and decreased alpha power and peak alpha frequency, together with an increased alpha coherence, as suggested by previous findings (Begić et al., 2001; Imperatori et al., 2014; Lee et al., 2014). In addition to the subjective self-reported symptoms and perception, the EEG analysis would objectivize the evaluation of the operators' condition. Providing an objective and, hopefully, a more sensitive outcome would have important social and professional implications, as exposure to stress may result in physical and mental disorders.

The aim of the present study was to evaluate the impact of severe stress exposure on healthcare professionals who have worked in direct contact with patients with COVID-19 after the pandemic “first wave” (Spring 2020). More specifically, we were interested in investigating potential changes in cortical activity, cognitive performances, the insurgence of burnout, and sleep quality. A quantitative evaluation of these items was therefore implemented and administrated two times through dedicated tests in order to detect possible consequences of severe stress exposure not only immediately after the outbreak of the sanitary emergency but also after a few months. This would allow the authors to draw a comparison between acute and recovery phases. Finally, as a stressful situation sets off a chain of physical reactions, such as a temporary acceleration of breathing and heart rate (Muraoka et al., 1998; Tan et al., 2011; Kim et al., 2018; Bustamante-Sánchez et al., 2020), we also aimed to assess if the two aforementioned parameters might provide additional and useful information.

## Materials and Methods

This single-center, observational longitudinal study was conducted at the Neurophysiopathology Unit of Fondazione IRCCS Ca' Granda Ospedale Maggiore Policlinico of Milan, Italy, in collaboration with Politecnico di Milano for data analysis. The protocol of the study was drawn up in conformity with Good Clinical Practice norms of the European Union and with the current revision of the Declaration of Helsinki and was approved by the Research Ethics Board of the IRCCS Ca' Granda Ospedale Maggiore Policlinico (439_2020).

### Subjects

The study cohort comprised healthcare professionals working at Fondazione IRCCS Ca' Granda Ospedale Maggiore Policlinico. The subjects were divided into two groups (Table 1), one of the frontline COVID-19 operators (FLCO) and one of the operators who worked in COVID-19-free units (CFO) during the outbreak of the pandemic (March-May 2020) and also after that time. Subjects were first enrolled among the staff of the Neuroscienze e salute mentale (Neuroscience and mental health) department, who were invited by e-mail to participate in the study and provided with information about the study goal and procedures. All participants volunteered and gave written consent. The inclusion criteria were the following: age <60 years, no neurological disorders, no post-traumatic syndrome disorder (assessed by interview before starting each of the two study sessions), no chronic therapy, and no leave of absence since the beginning of the pandemic. Healthcare workers were considered FLCOs when their entire shift required them to be in direct contact with patients with COVID-19 in order to have identical frequency and duration of exposure and proximity to patients.

**Table 1:** Description of the population.

		**Group**	
		**FLCO**	**CFO**
Sex (*n*)	Men	7	6
	Women	13	14
Age (years)		29.5 (26–34)	32 (27–47)
Employment (*n*)	Nurses	17	4
	Intermediate care technician	3	3
	Neurophysiopathology technician	0	3
	Administrative assistant	0	2
	Neurophysiopathology technician students	0	3
	Medical doctors or residents	0	5
Schooling (years)		16 (16–16)	16 (13–18.7)
Years of service (years)		5.5 (3–11)	4.5 (3–16.2)
Family members (*n*)		2 (1–2)	2 (2–2)

*CFO, operators who worked in COVID-19-free units; FLCO, COVID-19 frontline operators. Data are reported as mean (first quartile-third quartile)*.

### Experimental Design

All subjects were evaluated in the afternoon, at the end of their morning shift, and cognitive performances were tested resorting to the Italian version of the Montreal Cognitive Assessment (MoCA) and the Stroop Color and Word Test (SCWT), followed by the Pittsburgh Sleep Quality Index (PSQI) and the Maslach Burnout Inventory-General Survey (MBI-GS). Additionally, electroencephalogram (EEG) and electrocardiogram (EKG) signals were simultaneously recorded. Recordings and tests were carried out separately and administrated by the same operator. Both groups of subjects were evaluated two times: in a first session (18 May to 22 July 2020), immediately after the first epidemic wave, to evaluate stress in the acute phase, and in a second session (1 October to 12 November 2020), almost 6 months after, to evaluate possible chronic issues or recovery stress. All subjects did not take any naps during the morning work shift and did not take any psychostimulant drugs before the session. The caffeine intake was limited to one h before the acquisition of data.

### EEG and EKG Assessment

#### EEG

Brain activity was measured by EEG recording administered by qualified neurophysiologists in a quiet room, with all windows closed in order to minimize the noises coming from outside the building. It was recorded in the resting-state condition for 8 min, during which subjects were seated on a comfortable chair with their eyes closed. Subjects were instructed to stay awake and, whenever they were drifting into sleep, the neurophysiologist resorted to low audio input to help the subject keep vigilant (Jobert et al., 2013). The subjects were instrumented with 19 bridge silver and silver chloride electrodes fitted on a plastic prewired head cap and placed on the scalp according to the 10–20 system (namely: Fp1, Fp2, F3, F4, F7, F8, Fz, C3, C4, Cz, P3, P4, Pz, T3, T4, T5, T6, O1, and O2), with an electroconductive paste applied between the electrodes and the skin to guarantee optimal connection (Micromed System Plus Electroencephalograph, Galileo). The signals were recorded with a 512 Hz sampling frequency and filtered by an offline first order zero-phase Butterworth band-pass filter, with cutoff frequencies set, respectively, at 1.6 and 70 Hz to remove frequencies outside the range of interest. All segments of the signal affected by ocular, muscular, or other types of artifacts were removed manually and by means of independent component analysis and then a 50 Hz notch filter was applied to remove power line noise. After this pre-processing, the main EEG rhythms and dominant frequencies were extracted by spectral and connectivity analyses. Delta (1.6–4 Hz), theta (4–8 Hz), alpha (8–13 Hz), beta (13–30 Hz), gamma1 (30–50 Hz), and gamma2 (50–70 Hz) frequency bands were considered. Because we considered 0.5 Hz increments bins for all the bands, we had therefore acquired the 1.5 and 2 Hz data points, but the lower cutoff for the delta frequency band was 1.6 Hz (Jobert et al., 2013; Malver et al., 2014). For this reason, we performed a linear interpolation between the two discrete data points (1.5 and 2 Hz) with a window length of 2 s and 0.1 Hz increment to estimate the new data point of 1.6 Hz.

Welch's averaged modified periodogram method was implemented to determine the power spectral density (PSD) of the brain activity recorded by each electrode with a 2-s window length. The PSD curves' relative powers (i.e., the ratios between the area under the curve in each frequency band and the total area of the curve) were calculated to estimate how each rhythm contributes to the entire. Then, dominant frequencies (i.e., frequencies corresponding to the peaks of the power spectrum) were calculated first for the entire frequency range (Main Dominant Frequency) and then for the theta and alpha frequency bands (Peak Theta Frequency and Peak Alpha Frequency), being the main frequency bands related to stress and cognitive impairment (Klimesch, 1999; Begić et al., 2001; Hayes et al., 2012). Both parameters were computed for every single electrode as well as for each cerebral region of interest: i.e., central (C3, Cz, C4, F3, Fz, and F4 electrodes), posterior (T5, T6, P3, P4, Pz, O1 and O2 electrodes), occipital (O1 and O2 electrodes), and global (i.e., the entire scalp). Because the activity recorded by Fp1 and Fp2 electrodes was severely affected by residual ocular artifacts, which are mostly muscular artifacts, they were excluded from the analysis.

Besides spectral analysis, the connectivity analysis was also applied to detect the presence of temporal correlations among brain activities produced by spatially distant cortical areas. The coherence among activities recorded by electrodes located on the same cortical region but on opposite hemispheres was therefore calculated (Jorge et al., 2017). Interhemispheric coherence was firstly evaluated for theta, alpha, and beta rhythms, and then the entire frequency range was considered (Matlab R2020a and the plugin EEGLAB 2019.1 toolbox).

#### EKG

One electrode was placed on the left arm and a second one on the right arm in order to record a one-lead EKG for cardiac activity monitoring during EEG recording. The duration of the EKG recording was also 8 min. The number of heartbeats was obtained through the implementation of the Pan-Tompkins algorithm, a method that detects the QRS complexes in an EKG signal (Pan and Tompkins, 1985). Then, the standard deviation of R-R intervals (SDRR) was derived as a time-domain measurement of heart rate variability (HRV) (Tan et al., 2011; Järvelin-Pasanen et al., 2018; Kim et al., 2018). Finally, because the respiratory signals can be indirectly derived by the EKG signal, the respiratory sinus arrhythmia (RSA) method was implemented to calculate the breathing rate (Cysarz et al., 2008).

### Neuropsychological Assessment

All participants underwent a comprehensive neuropsychological assessment, including the Italian version of the Montreal Cognitive Assessment (MoCA), the Stroop Color and Word Test (SCWT), the Pittsburgh Sleep Quality Index (PSQI), and the Maslach Burnout Inventory-General Survey (MBI-GS).

#### MoCA Test

The test screens the global cognitive functioning to detect cognitive impairment and it is composed of six items, each focused on a specific cognitive domain: (1) memory [0–5 points]; (2) visuospatial ability [0–4 points]; (3) executive function [0–4 points]; (4) attention and working memory [0–6 points]; (5) language [0–6 points]; and (6) orientation in time and space [0–6 points]. A single score is assigned to each item according to the performance, and then, a total score is calculated by adding up all single scores and normalized according to the educational level of the subject, up to a maximum value of 30 points. The higher the total score, the better the cognitive performances are. Cognitive functions are considered normal with a total score of ≥15.51 points (Nasreddine et al., 2005; Smith et al., 2007; Santangelo et al., 2015).

#### SCWT

This is a neuropsychological test used to evaluate working memory and selective attention, processing speed, and cognitive flexibility, as cognitive processes are associated with the frontal lobe of the brain. The test assesses the ability to inhibit cognitive interference, which occurs when the processing of a stimulus feature affects the simultaneous processing of another attribute of the same stimulus (Stroop, 1935; Caffarra et al., 2002). The SCWT we administered is divided into three parts: reading, denomination, and interference. The SCWT was administered by means of physical cards and subjects were asked to read three different tables as fast as they could. Two of them represented the “congruous condition” in which participants are required to read names of colors (henceforth referred to as “color-words”) printed in black ink (W) and then name different color patches (C). Conversely, in the third table, named “color-word (CW) condition,” color words were printed in an inconsistently colored ink (for instance, the word “red” is printed in green ink). Thus, in this incongruent condition, participants were required to name the color of the ink instead of reading the word. The test was evaluated by counting the interference effect error (the number of mistakes/omissions made by the subject) and by the interference effect time (the time required to accomplish the test). Both the interference effect error and the interference effect time were obtained by subtracting the mean values of reading and denomination parts from the values of the third interference part with the formula: WC – [(W + C)/2], indicating the degree to which the person has control over interference. A participant has good cognitive performances with 0 or few mistakes and a very short time of execution.

### Questionnaires

#### PSQI

This is a self-rated questionnaire that evaluated the quality of sleep and its associated disturbances in the month preceding the test (Buysse et al., 1989). It is composed of 19 questions combined to assess the following seven items of sleep-related specific aspects: 1. subjective sleep quality; 2. sleep latency; 3. sleep duration; 4. sleep efficiency; 5. sleep disturbances; 6. use of sleep medications; and 7. daytime dysfunctions. Each item is scored between 0 (absence of disturbances) and 3 (serious problems associated with the specific sleep aspect). All scores are then summed together to obtain a global PSQI score, ranging between 0 and 21 points. The higher the total score, the worse the sleep quality is.

#### MBI-GS

This is a self-rated questionnaire used to assess the perceived burnout in the working environment (Maslach et al., 1996; Mealer et al., 2009), including healthcare professionals (Hallberg and Sverke, 2006) and social workers (Kim and Ji, 2009). The questionnaire asks participants 22 questions. The subject has to score each question with a number of points between 0 (never perceived) and 6 (felt everyday), depending on how often they feel the situation or emotion described. The questions are then combined into 3 main items that assess burnout: (1) emotional exhaustion [0–54 points]; (2) depersonalization [0–30 points]; and (3) personal accomplishment [0–48 points]. Each item is usually described in a qualitative way by using the terms low, moderate, or high. Higher scores of emotional exhaustion and depersonalization, together with low scores of personal accomplishment, are indicators of burnout.

### Statistical Analysis and Power Analysis

After computing all the parameters from each test, a statistical analysis was performed to determine the presence of statistically significant differences between data according to two factors: groups (FLCO vs. CFO) and session times (acute phase vs. chronic phase). The Kolmogorov-Smirnov test was applied to verify the distribution of the datasets and data were not normally distributed. For this reason, non-parametric tests were used for the statistical analysis of data. The Kruskal–Wallis and the Friedman tests were performed: the former was used to compare FLCO and CFO groups for each session; the latter was used to compare the two sessions within each group. A significance level equal to 5% was considered significant for both tests. The statistical analysis was performed in MatLab 2020a (MathWorks, Natick, MA). We considered theta relative power in the global region as our primary outcome. We could not find relevant published data to base a sample size calculation on, as theta relative power in the global region was never performed before on this kind of operator. For this reason, we ran a pilot study on 8 operators (4 FLCO: 3 women, median age: 34.5 years; 3 nurses; 1 intermediate care technician and 4 CFO: 3 women, median age: 40.2 years; 3 nurses; 1 intermediate care technician) to compute the mean (FLCO: 11.4; CFO: 14.8) and standard deviation (FLCO: 3.4; CFO: 2.1) of theta relative power in the global region. The difference between these two independent means provided an effect size of 1.203 that, with a type-1 error probability α = 0.05, a power (1-β, with β being type-2 error probability) of 0.95, and an allocation ratio of 1, resulted in a sample size of 38 subjects (19 FLCO and 19 CFO). The power analysis was performed in G^*^Power 3.1.9.4 software.

## Results

### Subjects

A total of 40 healthcare professionals volunteered to participate in the study: 20 frontline COVID-19 operators (FLCO) and 20 operators who worked in a COVID-19-free unit (CFO). The main characteristics of both groups are summarized in Table 1.

Because of the huge amount of data collected and in order to make the results more readable, we decided to report only statistically significant results with clinical implications, in CFO and FLCO groups at the first session and/or in CFO and FLCO groups in the second session and/or within the CFO group between the two sessions and/or within the FLCO group between the two sessions. All the other results of this exploratory analysis can be found in Supplementary Material.

### EEG and EKG

**Figure 1:**
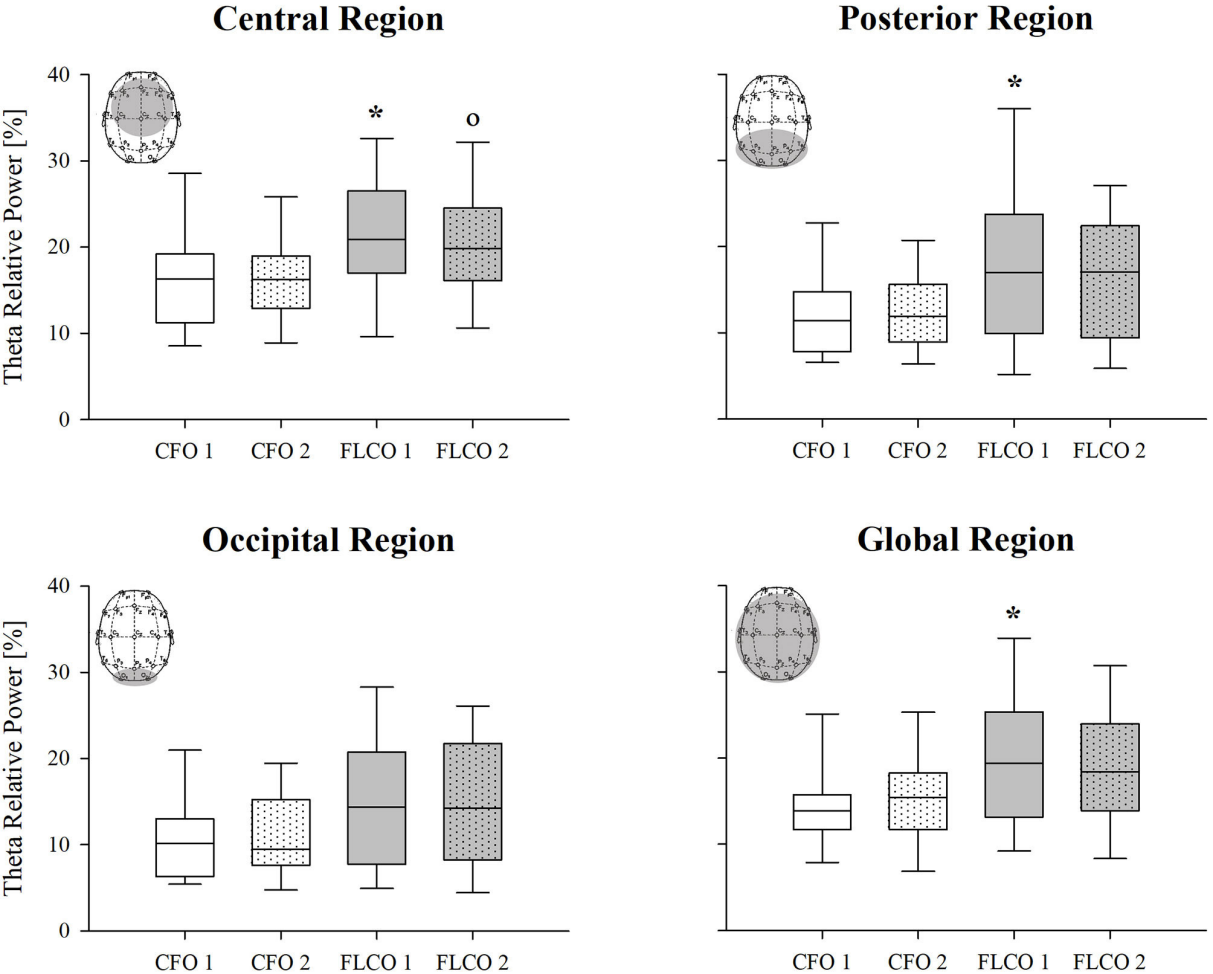
A box-and-whisker plot representing the median (line within the box), the interquartile range (length of the box), the 90th and 10th percentiles (whiskers above and below the box) of the electroencephalographic derived theta relative power in central (top left panel), posterior (top right panel), occipital (bottom left panel), and global regions (bottom right panel) in operators who worked in COVID-19-free wards and departments (CFO, white) and in frontline COVID-19 operators during the pandemic (FLCO, gray) during the first (1) and the second sessions (2). *p < 0.05 CFO1 vs. FLCO1; °*p* < 0.05 CFO2 vs. FLCO2.

**Figure 2:**
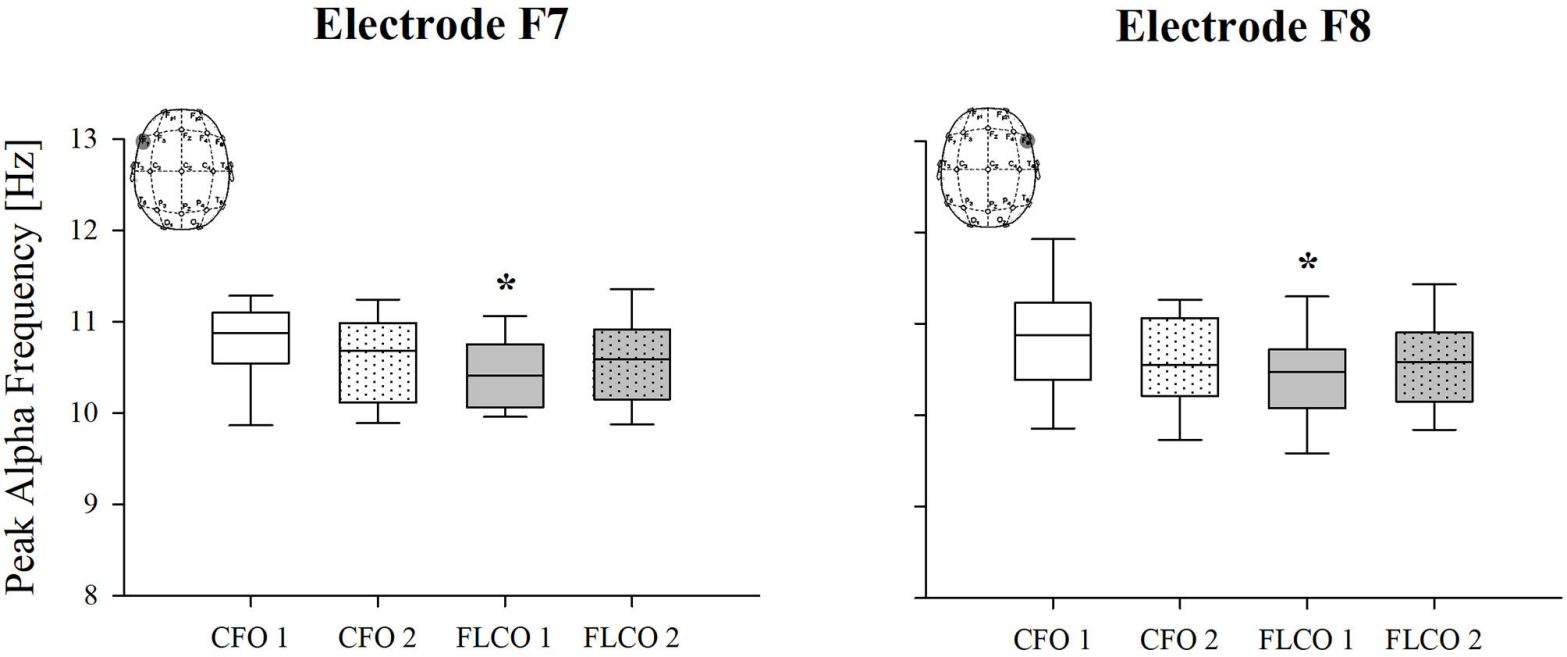
A box-and-whisker plot representing the median (line within the box), the interquartile range (length of the box), the 90th and 10th percentiles (whiskers above and below the box) of the electroencephalographic derived peak alpha frequency (PAF) in F7 (left panel) and F8 electrodes (right panel) in operators who worked in COVID-19-free wards and departments (CFO, white) and in frontline COVID-19 operators during the pandemic (FLCO, gray) during the first (1) and the second sessions (2). *p < 0.05 CFO1 vs. FLCO1.

Among the power spectral density curves relative powers, significance was found only for the theta frequency band. In the first session, the theta relative power of FLCO was significantly higher than CFO when considering the central, posterior, and global regions. The theta relative power of FLCO also remained higher in the second session but only in the central region (Figure 1). The Peak Alpha Frequency was systematically lower in FLCO in the first session for electrodes F7 and F8 (Figure 2).

**Figure 3:**
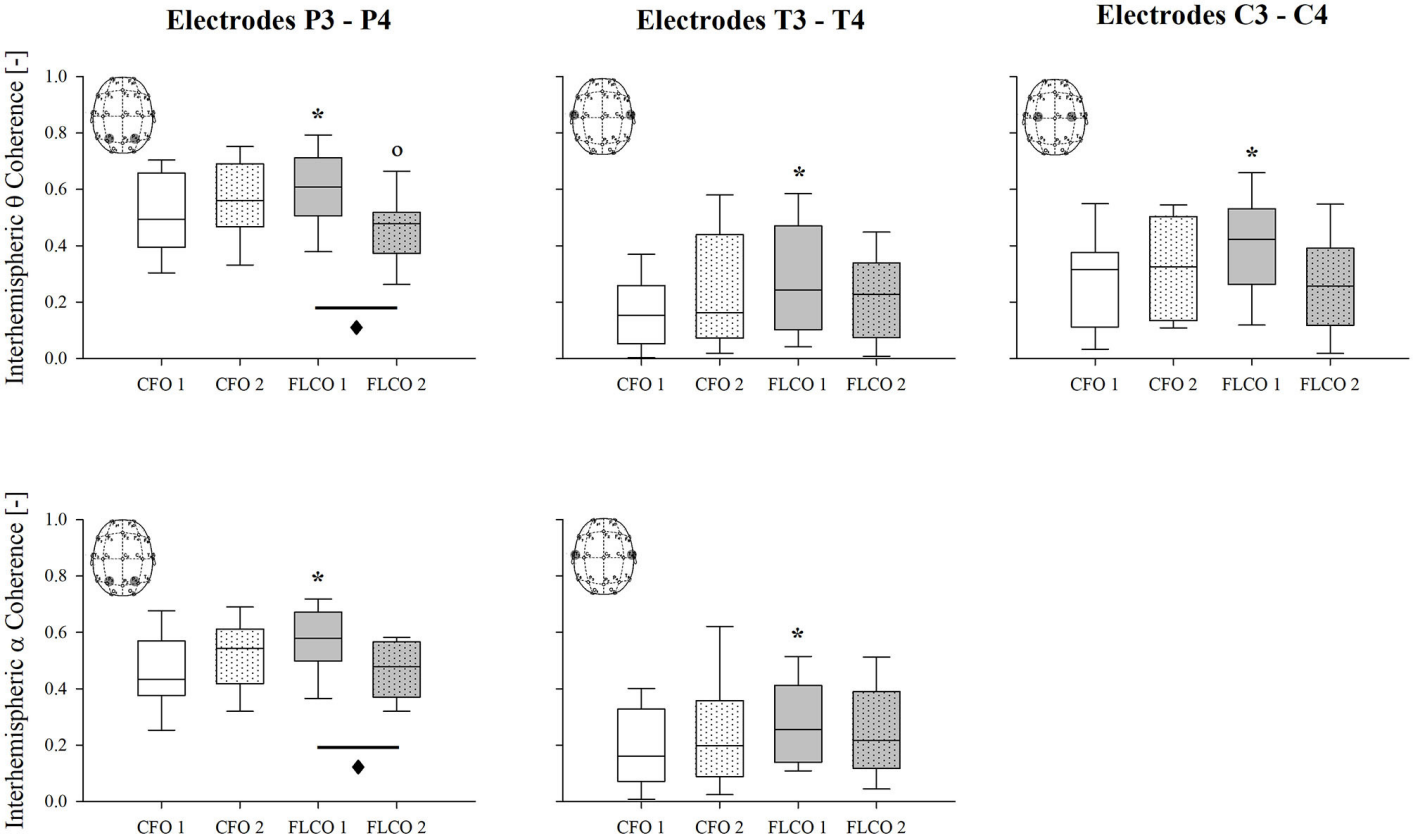
A box-and-whisker plot representing the median (line within the box), the interquartile range (length of the box), the 90th and 10th percentiles (whiskers above and below the box) of the electroencephalographic derived interhemispheric theta coherence in P3-P4 (top left panel), T3-T4 (top central panel), C3-C4 (top right panel) electrodes and interhemispheric alpha coherence in P3-P4 (bottom left panel) and T3-T4 electrodes (bottom central panel) in operators who worked in COVID-19-free wards and departments (CFO, white) and in frontline COVID-19 operators during the pandemic (FLCO, gray) during the first (1) and the second sessions (2). *p < 0.05 CFO1 vs. FLCO1; °*p* < 0.05 CFO2 vs. FLCO2; ♦*p* < 0.05 FLCO1 vs. FLCO2.

A significantly higher interhemispheric coherence of both the alpha and theta rhythms was found in FLCO in the first session between two couple of electrodes, P3-P4 and T3-T4, although they were both significantly reduced in the second session between electrodes P3-P4. When considering electrodes C3-C4, only the interhemispheric theta coherence was higher in FLCO in the first session (Figure 3).

During the first session, differences were also found in the EKG with FLCO being characterized by higher median heart rate (FLCO: 78.24 bpm; CFO: 71.1; *p* = 0.024) and consequently reduced R-R interval (FLCO: 0.767 s; CFO: 0.853 s; *p* = 0.0207). However, median HRV did not differ between the two groups during the first (FLCO: 83.1; CFO: 78.1; *p* = 0.705) and second (FLCO: 56.7; CFO: 60.1; *p* = 0.955) sessions, although it changed between the two sessions in both FLCO (*p* = 0.011) and CFO (*p* = 0.025) groups.

### Cognitive Tests and Questionnaires

**Figure 4:**
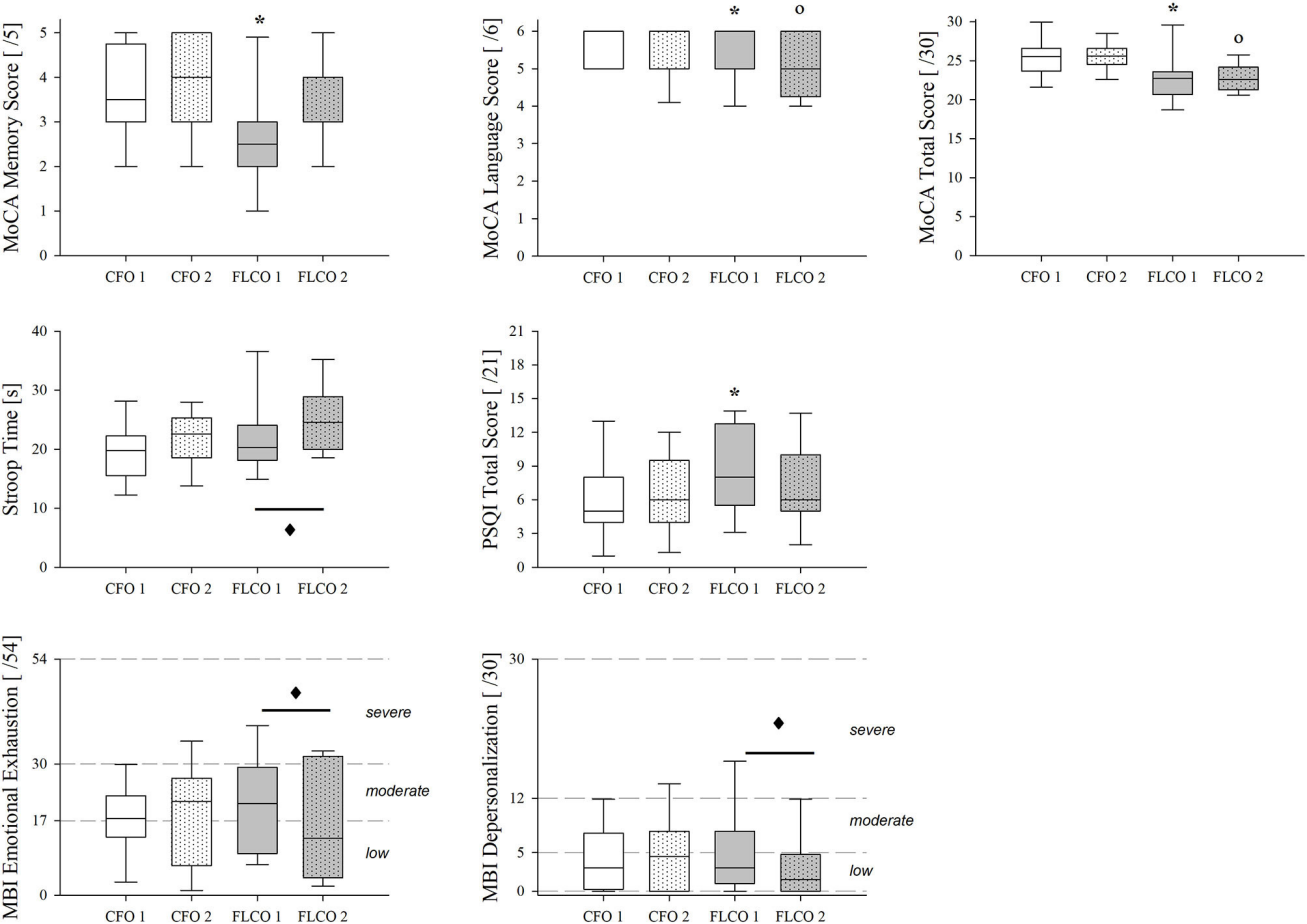
A box-and-whisker plot representing the median (line within the box), the interquartile range (length of the box), the 90th and 10th percentiles (whiskers above and below the box) of cognitive tests, and surveys results: MoCA memory score (top left panel), MoCA language score (top central panel), MoCA total score (top right panel), Stroop Color and Word Test time duration (middle left panel), Pittsburgh Sleep Quality Index Total Score (middle central panel), MBI-GS Emotional Exhaustion (bottom left panel), and depersonalization (bottom central panel) in operators who worked in COVID-19-free wards and departments (CFO, white) and in frontline COVID-19 operators during the pandemic (FLCO, gray) during the first (1) and the second sessions (2). *p < 0.05 CFO1 vs. FLCO1; °*p* < 0.05 CFO2 vs. FLCO2; ♦: *p* < 0.05 FLCO1 vs. FLCO2.

The MoCA test scores were lower in the FLCO group at the first session in language and memory items as well as in the total score. The language item score and the total score still remained lower in the second session (Figure 4), as well as executive functions and attention/working memory items.

The time duration of the Stroop Color and Word Test changed only in the FLCO group, increasing during the second session (Figure 4).

A higher Pittsburgh Sleep Quality Index total score characterized the FLCO group in the first session of measurements (Figure 4).

According to the Maslach Burnout Inventory-General Survey, the emotional exhaustion and the depersonalization in FLCO group were reduced between the first and second sessions (Figure 4).

## Discussion

To our best knowledge, none of the published studies on the effects of severe stress exposure in healthcare professionals working in hospitals during the COVID-19 pandemic was based on the evaluation and analysis of the electrical activity of the subjects' brains. Although our pilot study was conducted on a relatively small population, we found a significantly altered pattern in frontline COVID-19 operators compared to those who worked in COVID-19-free units. The potential abnormalities were the following: higher theta relative power, lower peak alpha frequency, higher interhemispheric coherence of both alpha and theta rhythms, and higher heart rate. Frontline COVID-19 operators were also characterized by lower MoCA test scores and higher Pittsburgh Sleep Quality Index scores. This reduction in cognitive performance and sleep quality may be attributed to stress exposure, but direct testing is still needed.

We might infer such differences to the additional stress of working directly with patients affected by a—till then—unknown virus.

### EEG Spectral Analysis

We observed that theta power was higher in the FLCO group on the entire scalp. However, the main significant differences were detected in electrodes located in the central region, in particular on the central axis and on the right hemisphere (data not shown), which is involved in the regulation of emotions (Hugdahl, 2005). Theta band is associated with emotions and, therefore, variations of this specific band indicate alterations in the emotion field. Thus, theta power is an objective parameter to assess those alterations, aside from the subject reports and perceptions (Zhang et al., 2012, 2013; Kraus et al., 2020). In addition, theta power is easily calculated and highly reproducible in different studies. For all the above reasons, it was beneficial to include theta power in our analysis.

As expected, alpha was the dominant rhythm of the EEG signals, because cortical activities were recorded in a resting state condition with closed eyes. However, in our pilot study, it did not significantly differ between the two groups. This is in contrast with previous studies showing that a decreased alpha rhythm is associated with stress and stress-related disorders in combat veterans with post-traumatic stress disorder (Begić et al., 2001).

Instead, the peak alpha frequency significantly differed, being lower in the FLCO group's activity recorded by electrodes F7 and F8. Previous studies showed lower PAF to be associated with worse cognitive performances, especially memory skills (Klimesch, 1994, 1999). Therefore, PAF also seems to be a promising indicator of reduced cognitive function in healthcare operators, but this must be confirmed by future dedicated studies.

Both the significances in the relative powers and in the peak frequency were detected in the first measurement, while the differences between the two groups appeared reduced in the second one.

We can speculate about the mechanism behind these results. The increase in the spectral power of the theta rhythm and the reduction of the peak alpha frequency parameters are simultaneously modified secondary to exposure to high levels of stress (Kraus et al., 2020). The mental fatigue induced by such high attentional and cognitive demands might have played a central role. Indeed, mental fatigue is the main cause of reduced alertness and may trigger fatigue, irritability, and loss of motivation (Tanaka et al., 2012).

Increased theta frequencies, particularly in the frontal-central regions, may be caused by sleepiness, which might result from excessive workloads. Furthermore, a large literature links increased theta rhythm to conditions characterized by strong emotional (Zhang et al., 2012, 2013) and visual impact (Cavallaro et al., 2010), as well as to pathological conditions such as major depression, panic attacks, and generalized anxiety (Begić et al., 2001; Aftanas et al., 2003).

### EEG Connectivity Analysis

Interesting results came also from the connectivity analysis. A similar trend was found for almost all couples of electrodes: with the exception of frontal F3-F4 electrodes, the FLCO group showed higher coherence values in the first measurement and lower values in the second measurement.

The most important differences were detected in electrodes T3-T4 and P3-P4 for the theta and alpha bands. Although the interpretation of connectivity analysis is still debated, we can speculate it to be a potential index of stress since it was already associated with stress in healthy subjects, although assessed by different parameters (Alonso et al., 2015; Khosrowabadi, 2018) and used to determine the presence and the severity of post-traumatic stress disorder (Modarres et al., 2019).

### Tests and Questionnaires

The MoCA test total score, a rapid screening instrument for mild cognitive dysfunction, highlighted an overall worsening performance for the FLCO group in both sessions. The main functions involved were attention, working memory, visuospatial abilities, and language items. The subjects scored between 23 and 26, indicating a mild but noticeable cognitive decline in terms of mild forgetfulness, mild disorientation, and mild impairment in problem-solving. Interestingly, memory skill decreased in the first measurement but it improved in the second one, with a trend similar to the peak alpha frequency results. Similarly, the FLCO group was characterized by lower overall sleep quality in the first measurement as indicated by the Pittsburgh Sleep Quality Index, with improvements in the second session. The Maslach Burnout Inventory-General Survey scores detected a low to moderate level of burnout, with improvements in emotional exhaustion and depersonalization over time in the FLCO group. Finally, also the modifications that emerged from the Stroop Test confirmed slightly altered cognitive function in the FLCO group compared to the CFO group. Indeed, literature shows that mental fatigue resulting from a high level of stress exposure might determine a deficit of attention revealed by Stroop Test and highlighted by EEG modifications in terms of connectivity (Hassanin et al., 2021) and increased theta activity (Sánchez-Moguel et al., 2022).

Even if these results showed differences between the two groups, they did not reveal the presence of alarming sleep disorders, cognitive deficits, or levels of burnout.

### EKG Analysis

Finally, the heart rate was slightly higher in the first session of the FLCO group, although still within physiological values. As we know, a stressful situation sets off a chain of events: the body releases adrenaline, a hormone causing a momentaneous acceleration of breathing and heart rate (Muraoka et al., 1998; Tan et al., 2011; Kim et al., 2018; Bustamante-Sánchez et al., 2020). However, heart rate variability analysis did not show any difference between the two groups but only within the two sessions in both groups. In addition, breathing rates did not present any differences between groups or measurements.

### Strengths and Limitations

Taken together, from EEG signal analysis, we could claim that theta power, PAF, and coherence were the main cortical features affected by the stressful experience of healthcare professionals belonging to the FLCO group. In particular, significant differences were detected, respectively, in the central, frontal, and parieto-temporal areas, namely the regions where the structures involved in human stress response are located. This furtherly prove the solidity of our results.

Comparing EEG signals and cognitive tests to survey scores of, respectively, objective and subjective measurements, we noticed an interesting anomaly: despite the altered electroencephalographic and cognitive parameters, we found that the subjects did not perceive burnout. This might suggest that (1) subjective tools for sleep and burnout investigations might be weak in the assessment of the burden caused by a stressful situation, such as working on the frontline at the beginning of a severe pandemic or (2) EEG measurements might be more sensible and able to identify the reaction to severe stress exposure, anticipating the subjective sensation felt by the subjects. We are inclined toward the second option, with the FLCO participants not experiencing burnout while higher stress levels might be reasonably speculated (although not measured). In any case, introducing an objective evaluation to integrate the results of subjective surveys in future research is definitely worthwhile to identify early prodrome of possible issues.

It is important to underline that our subjects were deliberately selected among workers assigned continuously to full-time shifts and with no previous diagnosis of post-traumatic stress disorder. This is another strong point of our study, as it further confirms the sensitivity of the method in identifying early-altered signs secondary to severe stress exposure.

A potential limitation of this study might be the low number of subjects enrolled, particularly if compared to studies resorting to web-based surveys that were able to address large cohorts (Alonso et al., 2021; Gilleen et al., 2021). However, this was a pilot study aimed at verifying the potentiality of EEG as a means to identify the effects of severe stress exposure even in operators not yet affected by a severe stress syndrome, when no data could be found in the literature. Of course, the results should be further strengthened by enrolling larger cohorts of operators, but our preliminary data are encouraging since they already highlight important differences. We do not believe the mixed occupational roles (and hence small sample size for each role) of the CFO group to be confounding, as it reflected the real distribution of the working people at that specific time. Indeed, Lombardy was the epicenter of the first pandemic wave, characterized by a full lockdown of all activities not primarily related to the sanitary emergency (Villani et al., 2020). Some healthcare professionals were banned by the COVID-19 wards and therefore they could not be frontline COVID-19 operators by definition, while others (like the three intermediate care technicians of our FLCO group) were reassigned to a COVID-19 ward and needed to adapt to a new situation of full contact with patients. In that specific moment of uncertainty and dramatic situation in hospitals and clinics, the related changes were more likely a consequence of the proximity to severely ill patients rather than to a specific professional role.

Another limitation that goes beyond the design of the present study is the lack of a benchmark in terms of quantitative EEG analysis in the general population. Indeed, various factors of data analysis methods were proposed to contribute to several contradictory results. Some of these factors include a lack of standardized protocol, the brain region of interest, the stressor type, the recording duration, and proper EEG processing (Katmah et al., 2021).

We opted for quantitative electroencephalography, which distinguishes itself from clinical EEG by the application of complex mathematical approaches and computing scientific methods. Its quantitative nature enables a quantitative description of the waveforms of EEG signals feature extraction: analysis of specific frequency band and signal complexity, analysis of connectivity, and network analysis. The role of quantitative EEG is not necessarily an immediate diagnosis but provides additional insights that can be paired with other diagnostic evaluations in order to get the objective information necessary for a precise diagnosis, correct disease severity assessment, and specific treatment response evaluation (Livint Popa et al., 2020; Höller, 2021). This is the main strength and innovation provided by this pilot study.

Having operators who worked in COVID-19-free wards and departments as subjects is another strong point of the study, as it provides a useful control group to refer to. Although sex was equally distributed between groups but not within groups, we do not believe that this parameter might have played a role. As far as we know, EEG tracing does not differ between men and women.

The lack of direct measures (either subjective or objective) of stress or anxiety or depression is a major limitation. This was a pilot study mainly aimed at understanding the potential role of EEG in this kind of situation and cohort. The pilot study plays a role, among others, in evaluating the limits of the protocol in order to improve it upon the study design prior to a full-scale research project (In, 2017). We are aware that a direct measure of stress or anxiety or depression would be essential. In spite of this important issue, we believe that the results of the present pilot study highlight the potentiality of cortical activity as an objective means of assessment, with the potential use in occupational medicine. However, studies based on large cohorts have already shown how the pandemic has severely increased the level of stress among healthcare operators. Because there is evidence of an association between EEG modification and acute period stress (Katmah et al., 2021), we might infer the differences found between the two groups to the different levels of stress exposure at work. Finally, when we designed the study, we assumed that the second measurement would have happened in an improved epidemiological situation, both in Italy and worldwide, with less pressure on hospitals. Unfortunately, our second measurement coincided with the beginning of a second emergency crisis: therefore, it was not a recovery phase, as originally intended, but instead a second acute phase. Despite the circumstances, in the second measurement, the FLCO group showed a lower alteration, suggesting that some sort of chronic adaptive mechanism was in place in the FLCO group to cope with the prolonged stressful situation. For this reason, it would be useful to add a third data acquisition session at the very end of the sanitary emergency in order to detect potential symptoms of stress-related psychological disorders. Indeed, this type of illness usually does not arise immediately after the occurrence of an experienced traumatic event but after a given amount of time (Salazar de Pablo et al., 2020).

Additionally, this technique of evaluation secondary to stress exposure could be implemented beyond the medical field. For example, employees belonging to other working categories may have a lower threshold of stress tolerance compared to healthcare workers, and EEG could be therefore used as a new clinical evaluation tool for preventive interventions in occupational medicine to identify early signs of burnout. Indeed, occupational medicine is not based only on consolidated data but evolves according to emerging scientific evidence (Cristaudo, 2020). Stress and its negative impact have become a growing problem not only in daily lives but also in occupational medicine, as the different systems of the human body, such as the nervous, immune, cardiovascular, and gastrointestinal systems, are affected. Thus, objective evaluation and analysis of prolonged stress exposure are very important to detect stress-induced alteration to prevent significant health problems. The use of EEG evaluation could also be extended to patients suffering from COVID-19 since they are at risk of developing mental disorders like anxiety, depression, or post-traumatic distress syndrome (Rogers et al., 2020). Once again, this objective evaluation might have some important social and economic implications, considering that the virus has already infected over 230 million people worldwide (COVID-19 Map - Johns Hopkins Coronavirus Resource Center, 2022).

In conclusion, working in direct contact with patients with COVID-19 could be effectively considered an important cause of mental stress. One of the most likely negative outcomes of increased levels of stress, fatigue, and excessive workload in healthcare professionals is burnout. This might have important social and professional implications, as exposure to stress, especially for prolonged periods, may result in physical and mental disorders. Indeed, stress is a social determinant of health, and in order to avoid the risk of medical negligence, an unbiased, objective, and automated method for the diagnosis of stress should be adopted alongside the current self-reporting assessment methods.

## Data Availability Statement

The raw data supporting the conclusions of this article will be made available by the authors, without undue reservation.

## Ethics Statement

The studies involving human participants were reviewed and approved by Research Ethics Board of the IRCCS Ca' Granda Ospedale Maggiore Policlinico (439_2020). The patients/participants provided their written informed consent to participate in this study.

## Author Contributions

Conceptualization and methodology: MV and AL. Software and formal analysis: MM. Investigation: FM, FR, CD, and GA. Resources: RF, AA, and SB. Data curation: MM and AL. Writing original draft preparation and writing, reviewing, and editing: AL, MM, and MV. Supervision: RF, AA, and SB. Project administration: MV. All authors have read and agreed to the published version of the manuscript.

## Funding

Publication costs were funded by Grant Ricerca Corrente, Italian Ministry of Health.

## Conflict of Interest

The authors declare that the research was conducted in the absence of any commercial or financial relationships that could be construed as a potential conflict of interest.

## Publisher's Note

All claims expressed in this article are solely those of the authors and do not necessarily represent those of their affiliated organizations, or those of the publisher, the editors and the reviewers. Any product that may be evaluated in this article, or claim that may be made by its manufacturer, is not guaranteed or endorsed by the publisher.
