# Supplementary material for: EEG Evaluation of Stress Exposure on Healthcare Workers During COVID-19 Emergency: Not Just an Impression
Source: Front Syst Neurosci. 2022 Jul 18;16:923576. doi: 10.3389/fnsys.2022.923576 (PMC9339626; doi:10.3389/fnsys.2022.923576)
Supplement: Supplementary file 1 [file Table_1.DOCX]

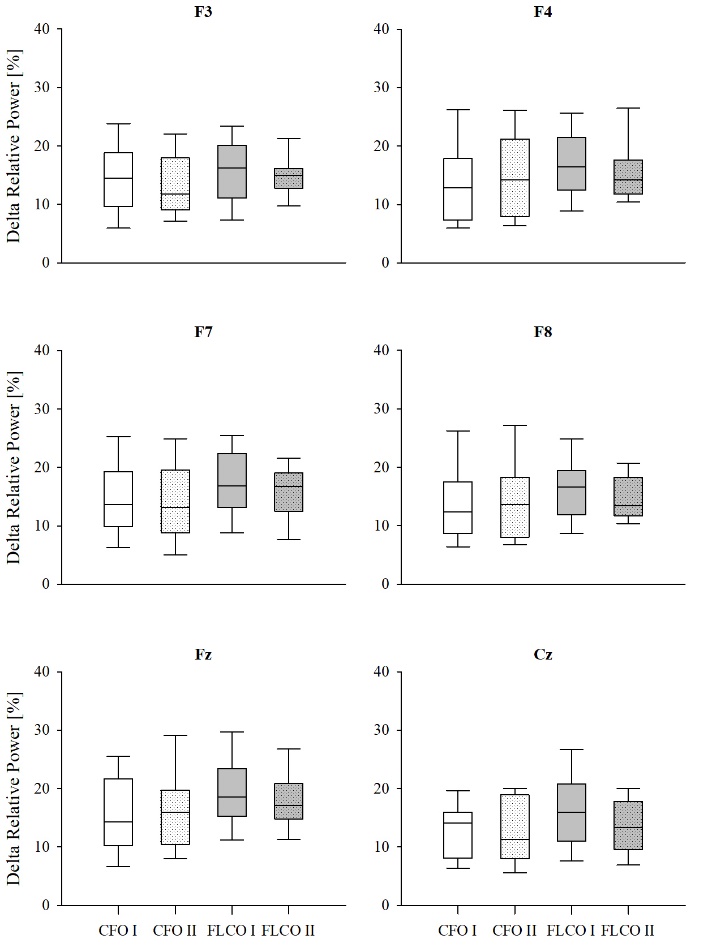

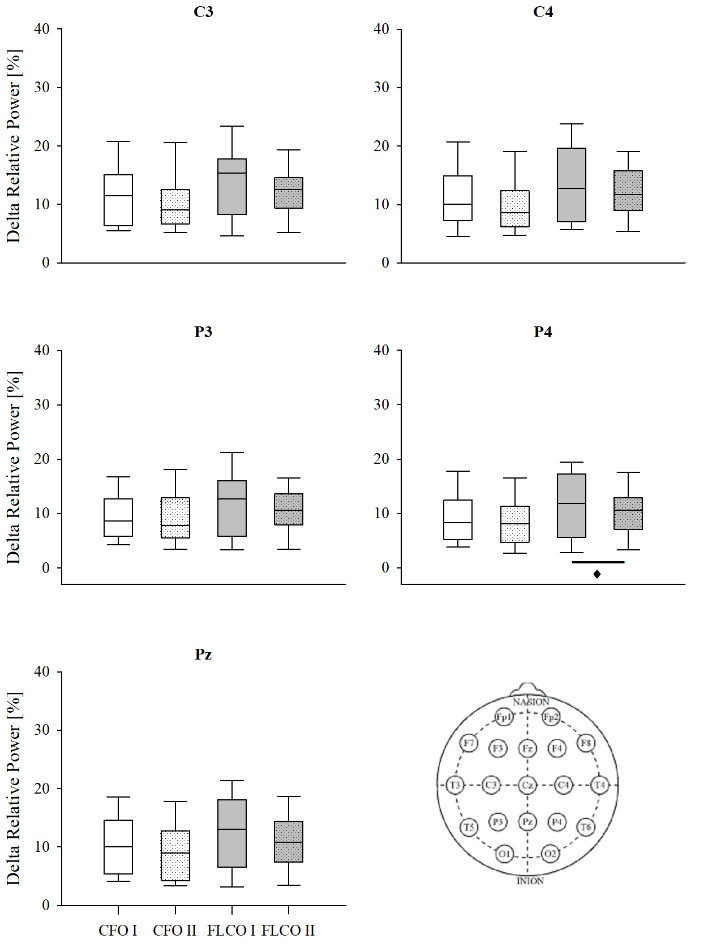

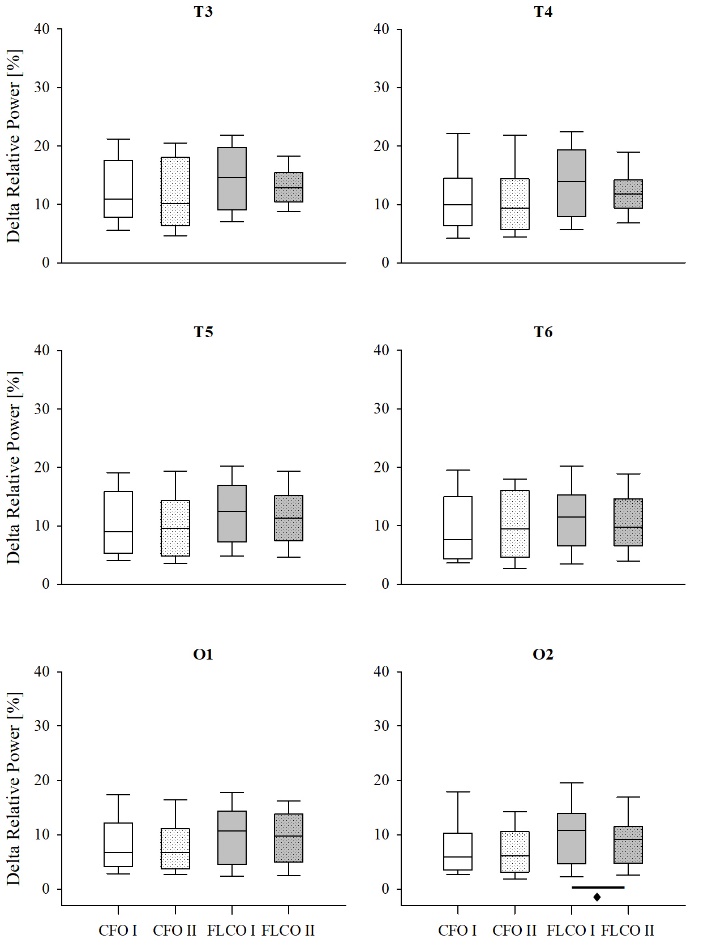


**Figure 1.** Box-and-whisker plot representing the median (line within the box), the interquartile range (length of the box), the 90th and the 10th percentiles (whiskers above and below the box) of the electroencephalographic derived Delta Relative Power in F3, F4, F7, F8, Fz, C3, C4, Cz, P3, P4, Pz, T3, T4, T5, T6, O1 and O2 electrode in operators who worked in COVID-19-free wards and departments (CFO, white) and in frontline COVID-19 operators during the pandemic (FLCO, grey) during the first (I) and the second session (II). *: p<0.05 CFO1 vs FLCO1; °: p<0.05 CFO2 vs FLCO2; ♦: p<0.05 FLCO1 vs FLCO2.


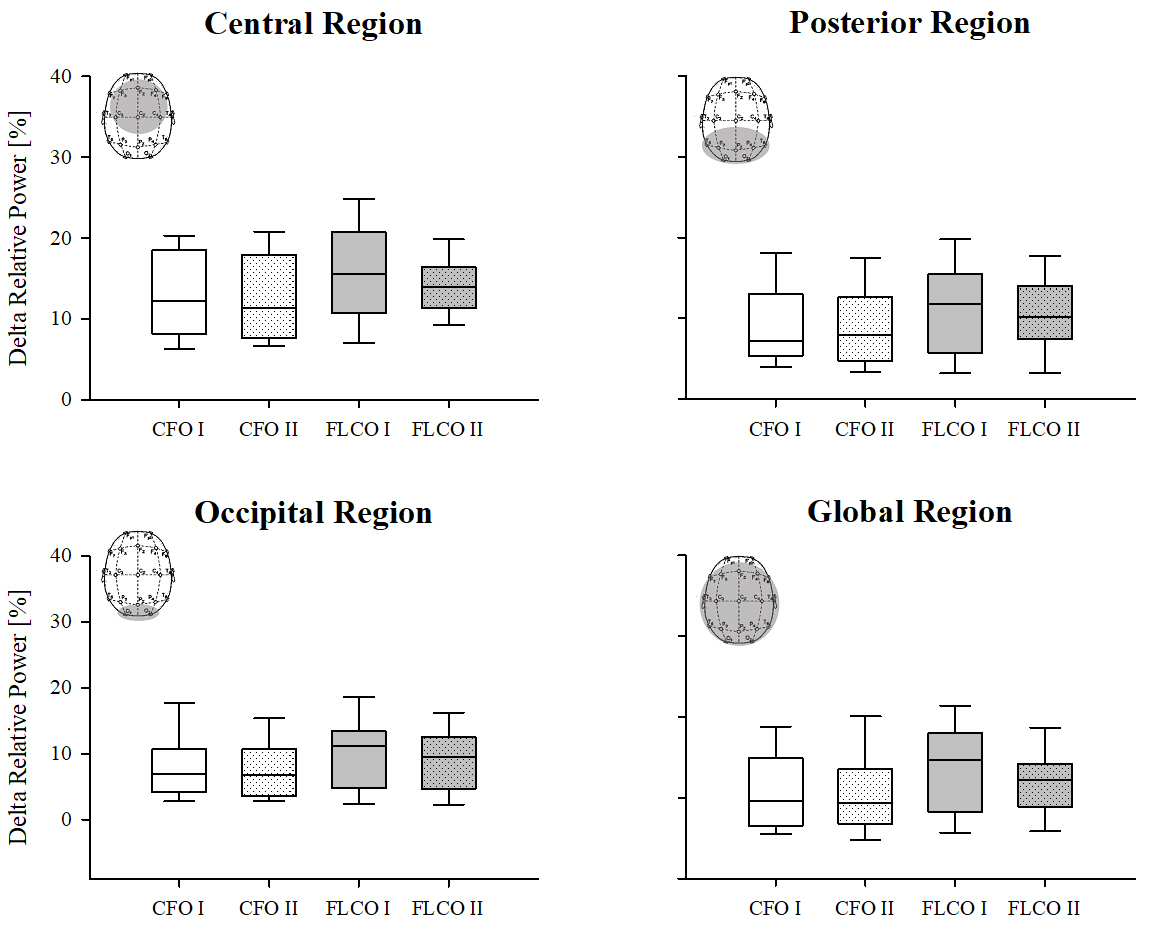


**Figure 2.** Box-and-whisker plot representing the median (line within the box), the interquartile range (length of the box), the 90th and the 10th percentiles (whiskers above and below the box) of the electroencephalographic derived Delta Relative Power in central (top left panel), posterior (top right panel), occipital (bottom left panel), and global regions (bottom right panel) in operators who worked in COVID-19-free wards and departments (CFO, white) and in frontline COVID-19 operators during the pandemic (FLCO, grey) during the first (I) and the second session (II). *: p<0.05 CFO1 vs FLCO1; °: p<0.05 CFO2 vs FLCO2; ♦: p<0.05 FLCO1 vs FLCO2.


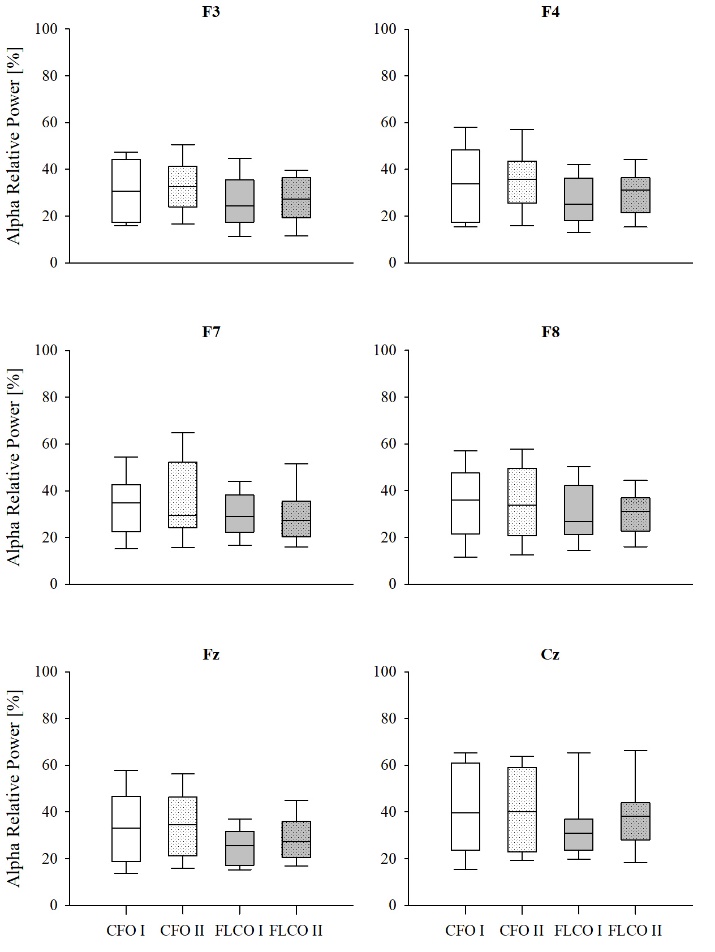

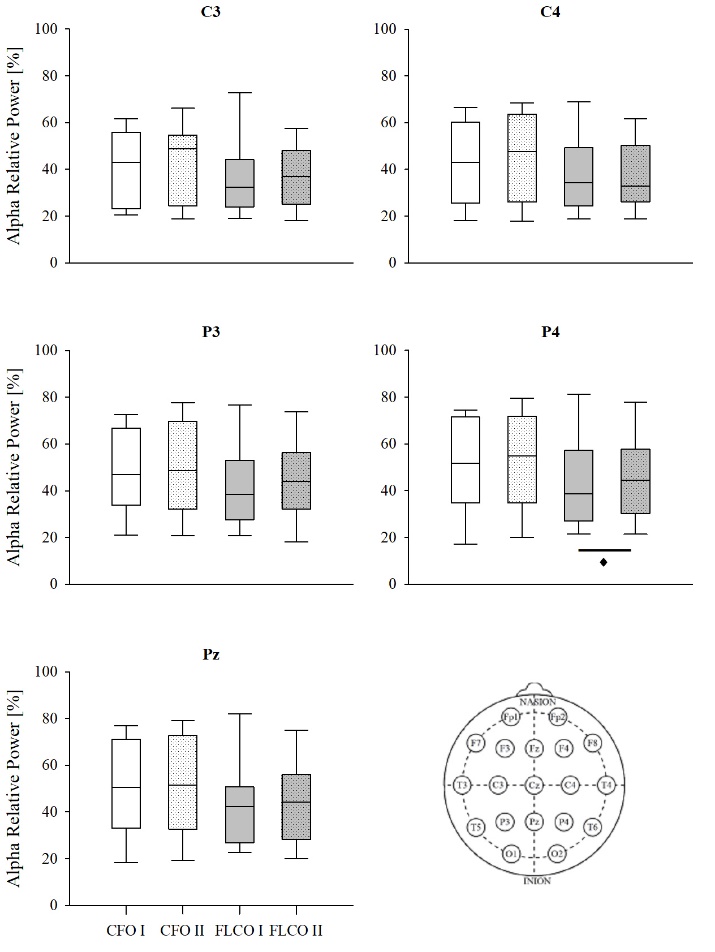

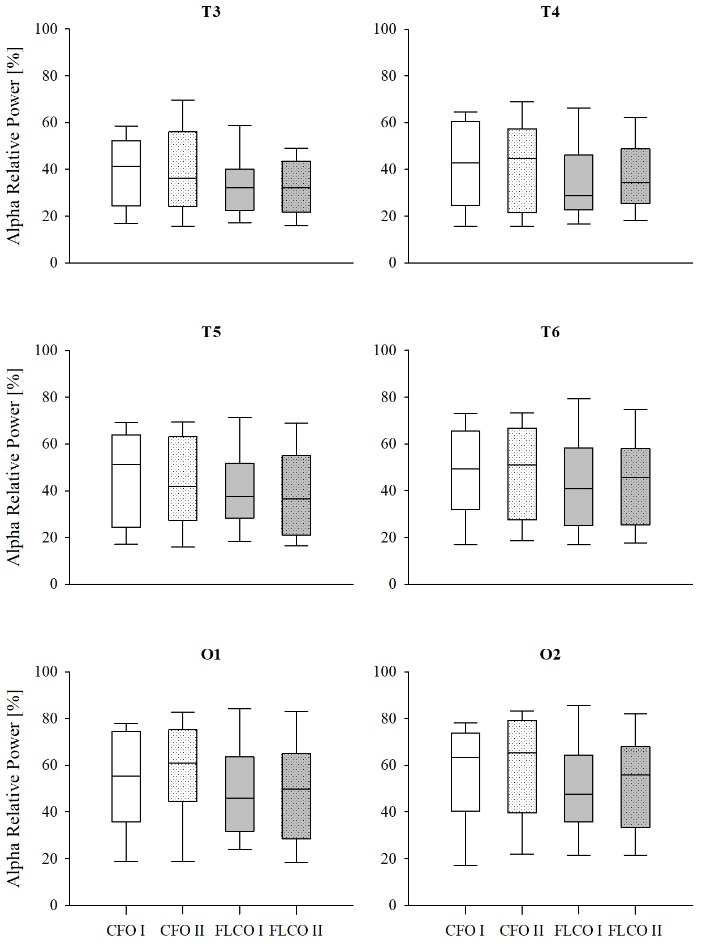


**Figure 3.** Box-and-whisker plot representing the median (line within the box), the interquartile range (length of the box), the 90th and the 10th percentiles (whiskers above and below the box) of the electroencephalographic derived Alpha Relative Power in F3, F4, F7, F8, Fz, C3, C4, Cz, P3, P4, Pz, T3, T4, T5, T6, O1 and O2 electrode in operators who worked in COVID-19-free wards and departments (CFO, white) and in frontline COVID-19 operators during the pandemic (FLCO, grey) during the first (I) and the second session (II). *: p<0.05 CFO1 vs FLCO1; °: p<0.05 CFO2 vs FLCO2; ♦: p<0.05 FLCO1 vs FLCO2.


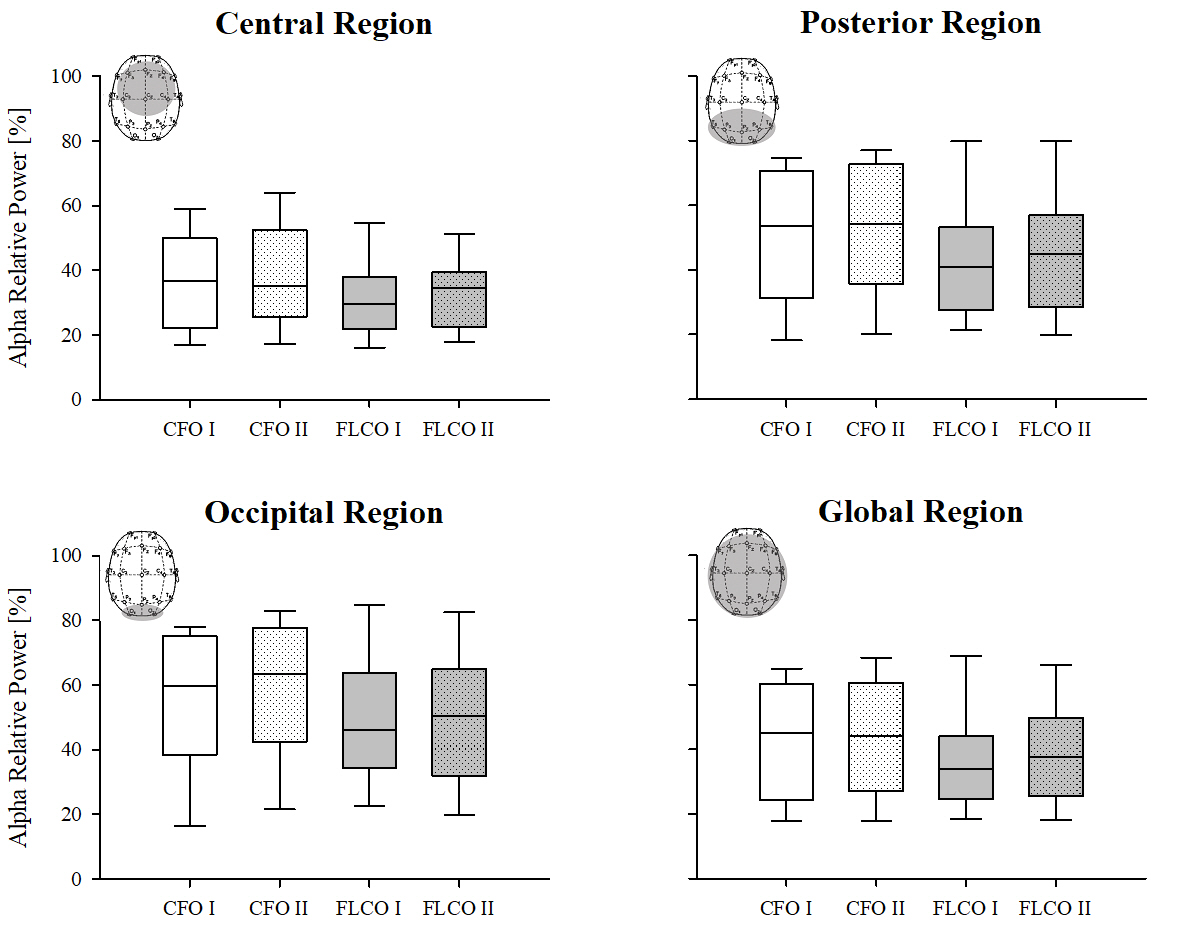


**Figure 4.** Box-and-whisker plot representing the median (line within the box), the interquartile range (length of the box), the 90th and the 10th percentiles (whiskers above and below the box) of the electroencephalographic derived Alpha Relative Power in central (top left panel), posterior (top right panel), occipital (bottom left panel), and global regions (bottom right panel) in operators who worked in COVID-19-free wards and departments (CFO, white) and in frontline COVID-19 operators during the pandemic (FLCO, grey) during the first (I) and the second session (II). *: p<0.05 CFO1 vs FLCO1; °: p<0.05 CFO2 vs FLCO2. ♦: p<0.05 FLCO1 vs FLCO2.


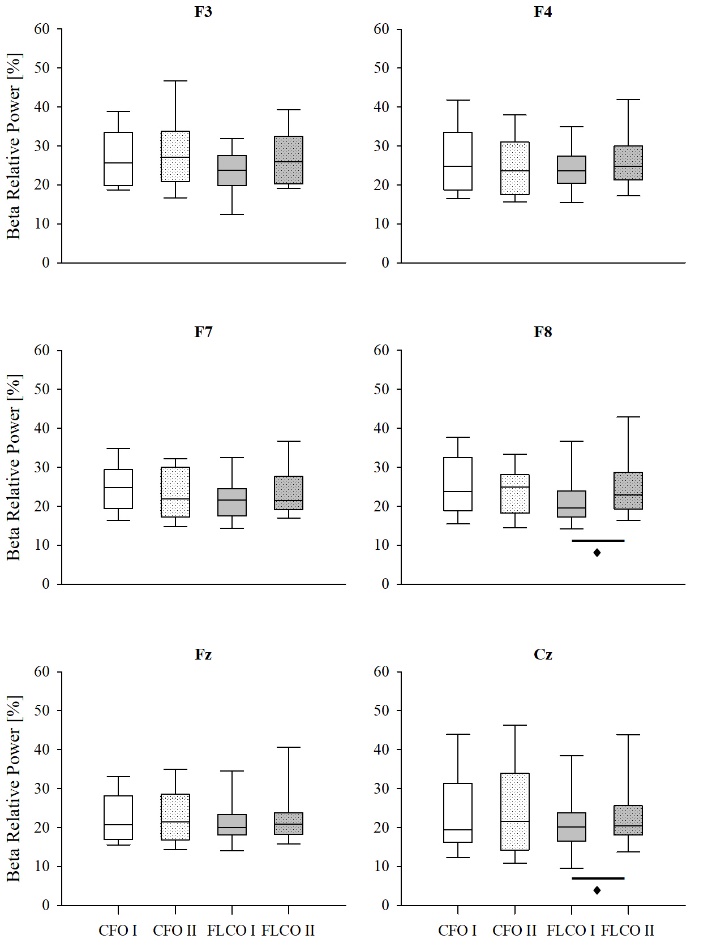

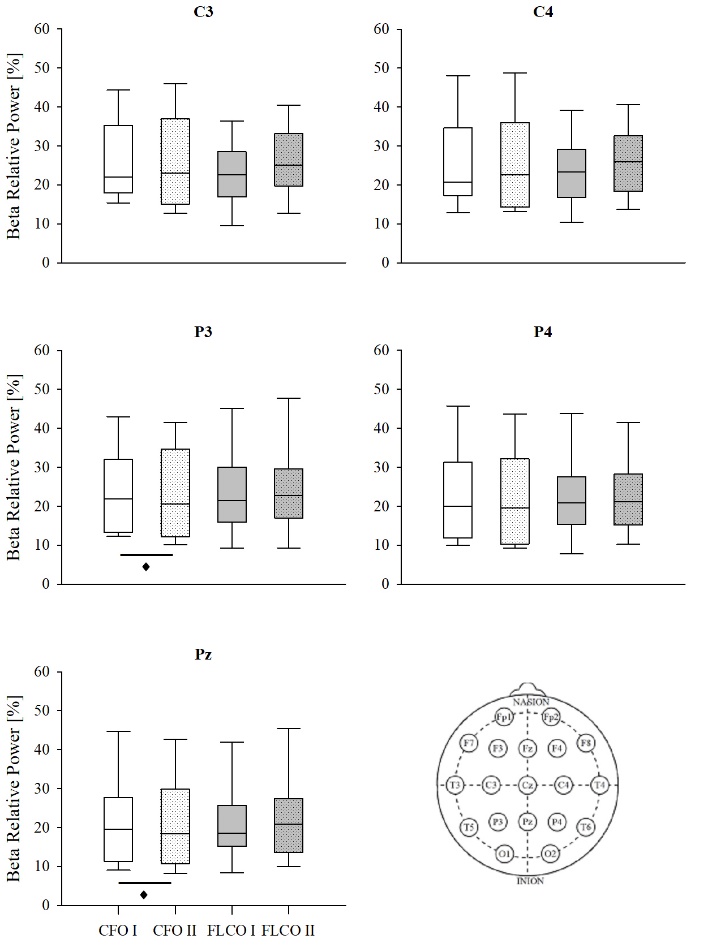

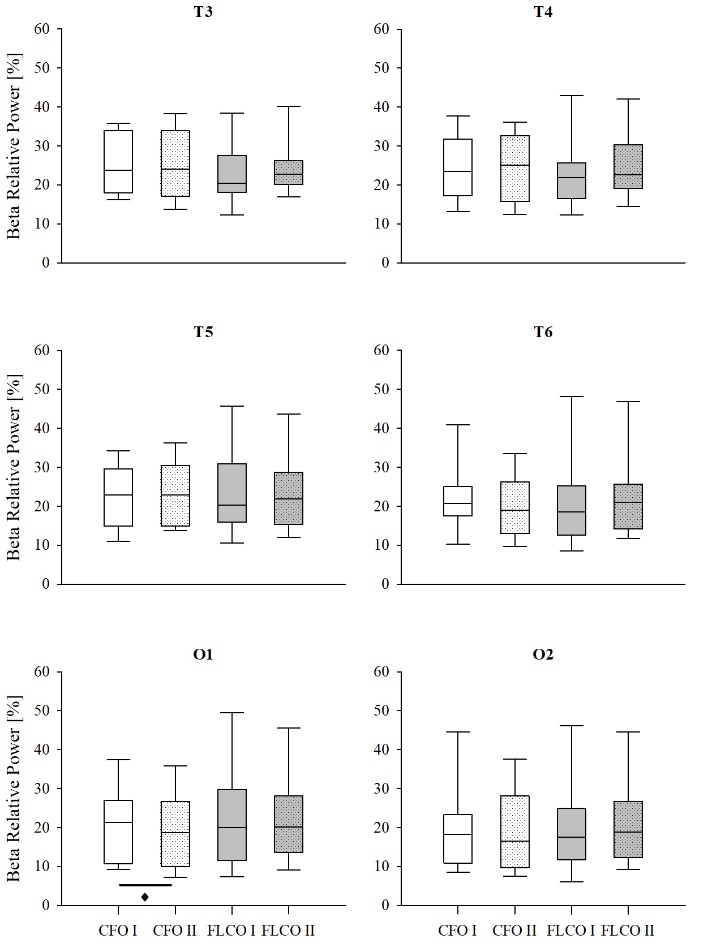


**Figure 5.** Box-and-whisker plot representing the median (line within the box), the interquartile range (length of the box), the 90th and the 10th percentiles (whiskers above and below the box) of the electroencephalographic derived Beta Relative Power in F3, F4, F7, F8, Fz, C3, C4, Cz, P3, P4, Pz, T3, T4, T5, T6, O1 and O2 electrode in operators who worked in COVID-19-free wards and departments (CFO, white) and in frontline COVID-19 operators during the pandemic (FLCO, grey) during the first (I) and the second session (II). *: p<0.05 CFO1 vs FLCO1; °: p<0.05 CFO2 vs FLCO2; ♦: p<0.05 FLCO1 vs FLCO2.


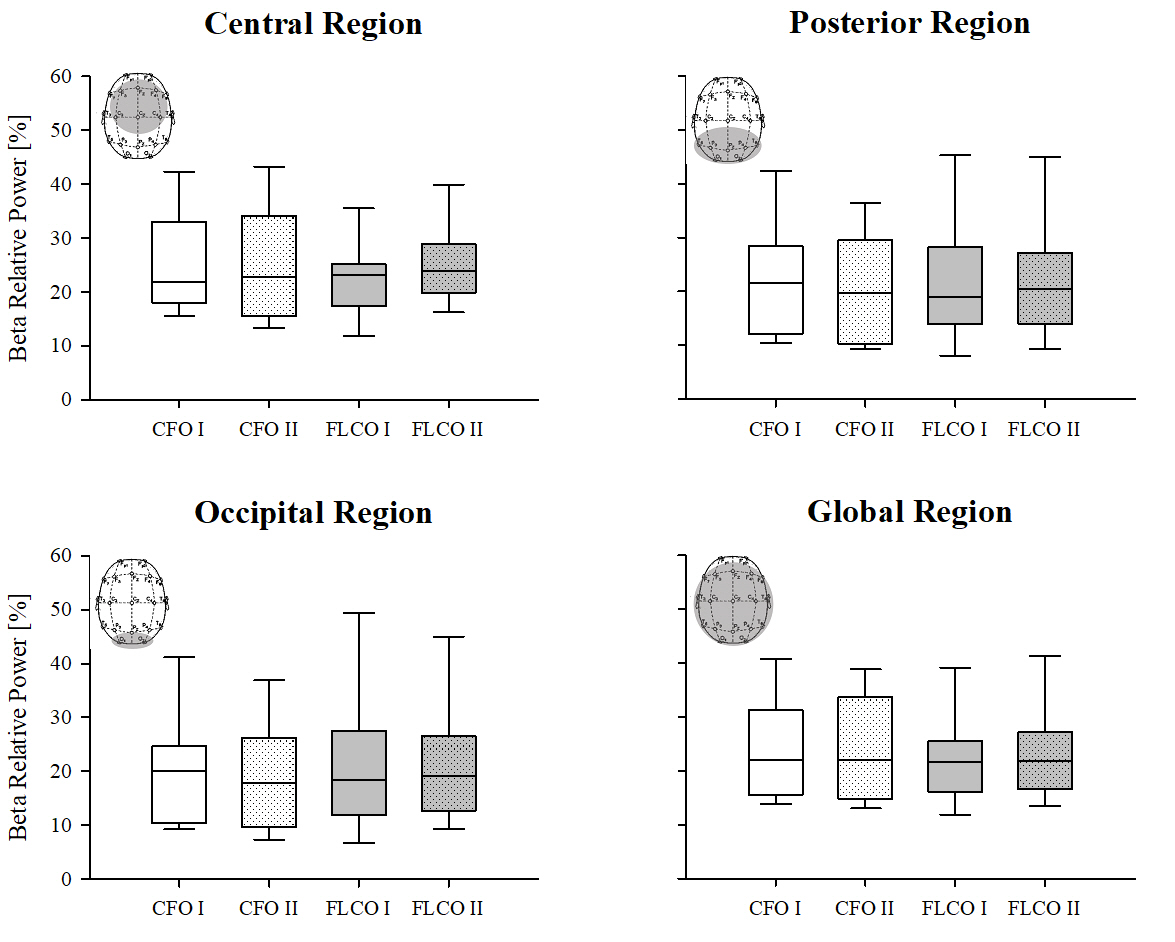


**Figure 6.** Box-and-whisker plot representing the median (line within the box), the interquartile range (length of the box), the 90th and the 10th percentiles (whiskers above and below the box) of the electroencephalographic derived Beta Relative Power in central (top left panel), posterior (top right panel), occipital (bottom left panel), and global regions (bottom right panel) in operators who worked in COVID-19-free wards and departments (CFO, white) and in frontline COVID-19 operators during the pandemic (FLCO, grey) during the first (I) and the second session (II). *: p<0.05 CFO1 vs FLCO1; °: p<0.05 CFO2 vs FLCO2. ♦: p<0.05 FLCO1 vs FLCO2.


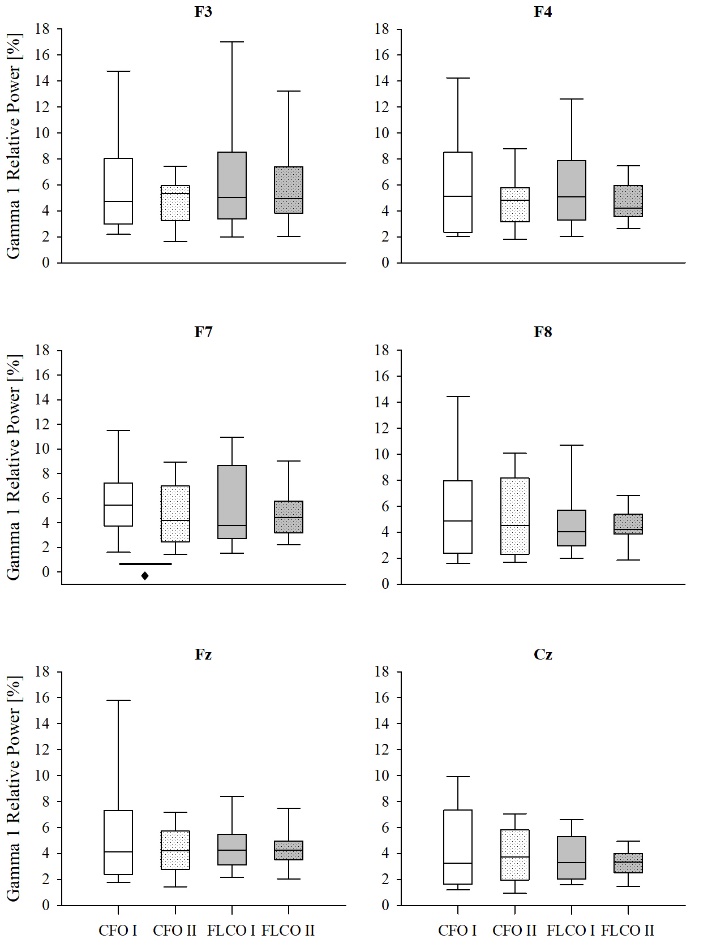

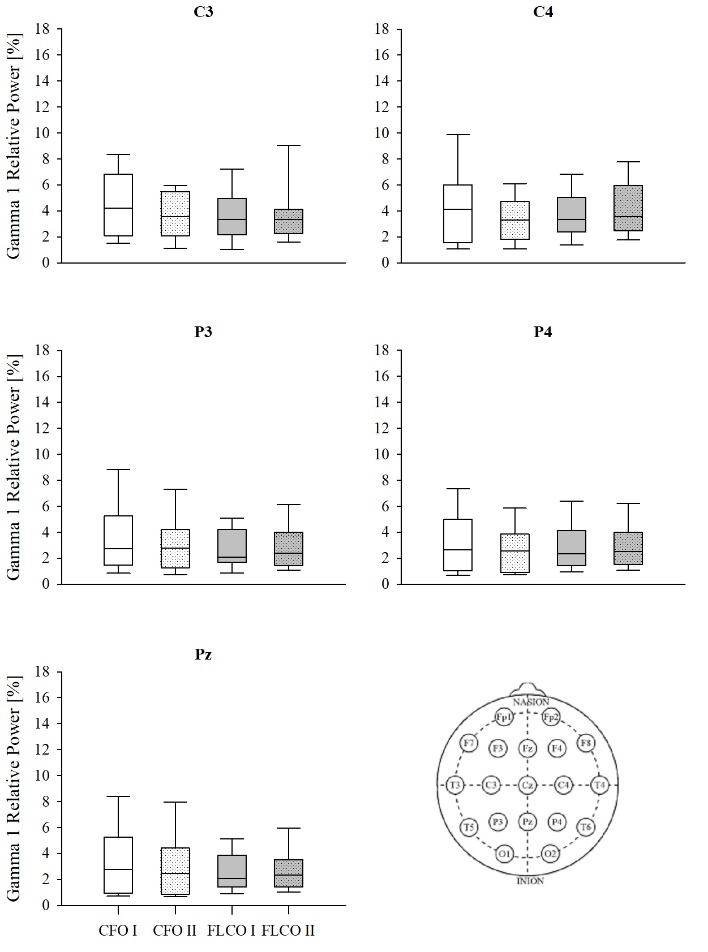

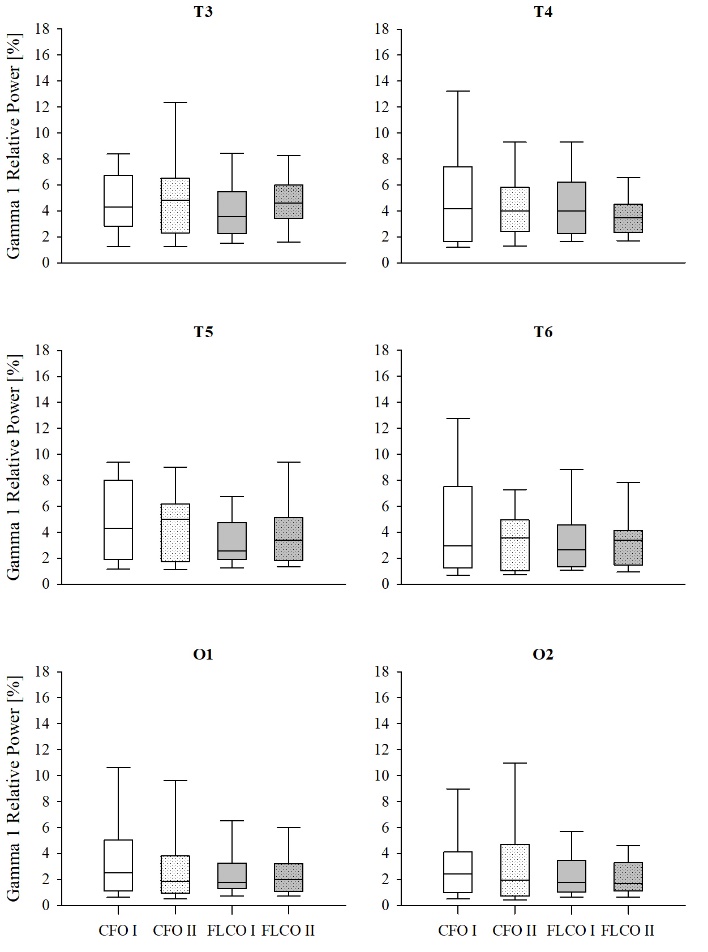


**Figure 7.** Box-and-whisker plot representing the median (line within the box), the interquartile range (length of the box), the 90th and the 10th percentiles (whiskers above and below the box) of the electroencephalographic derived Gamma 1 Relative Power in F3, F4, F7, F8, Fz, C3, C4, Cz, P3, P4, Pz, T3, T4, T5, T6, O1 and O2 electrode in operators who worked in COVID-19-free wards and departments (CFO, white) and in frontline COVID-19 operators during the pandemic (FLCO, grey) during the first (I) and the second session (II). *: p<0.05 CFO1 vs FLCO1; °: p<0.05 CFO2 vs FLCO2; ♦: p<0.05 FLCO1 vs FLCO2.


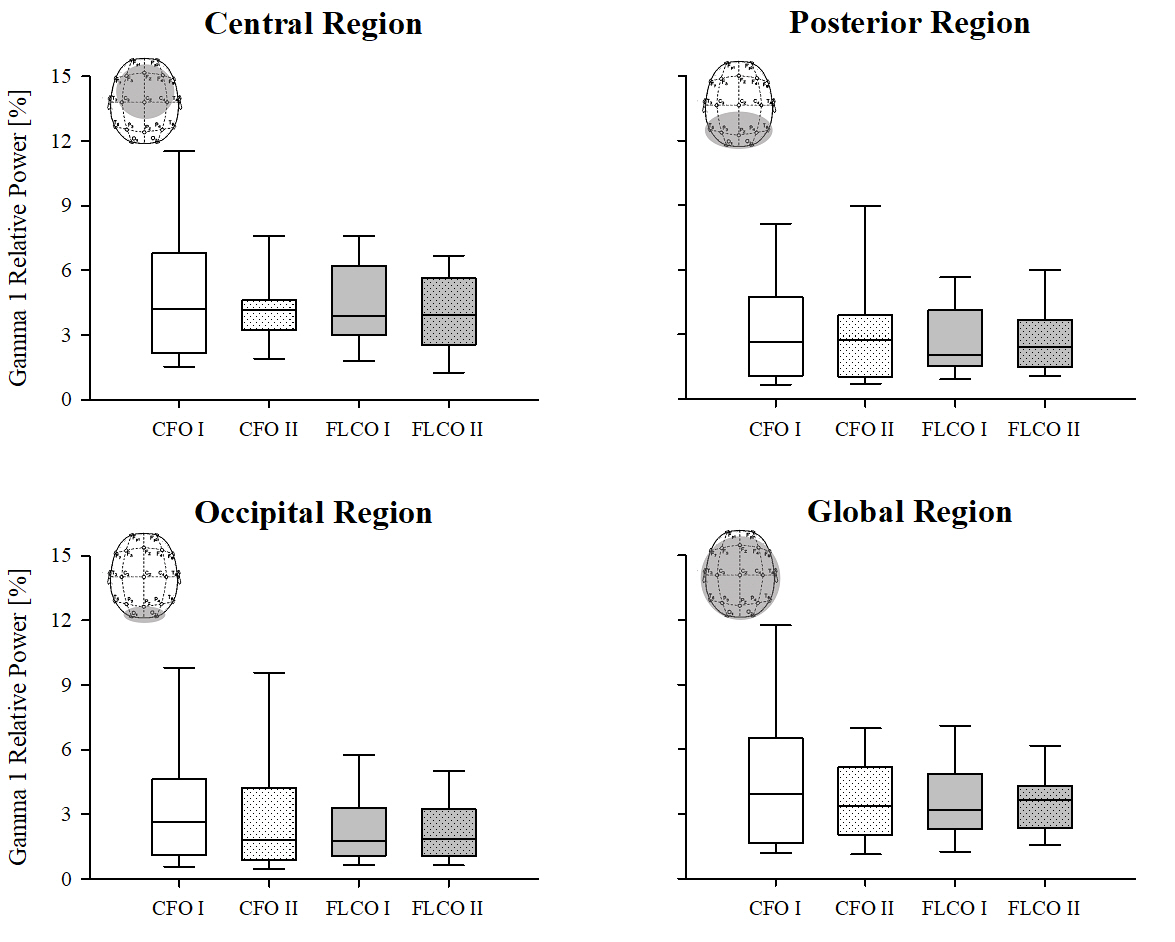


**Figure 8.** Box-and-whisker plot representing the median (line within the box), the interquartile range (length of the box), the 90th and the 10th percentiles (whiskers above and below the box) of the electroencephalographic derived Gamma 1 Relative Power in central (top left panel), posterior (top right panel), occipital (bottom left panel), and global regions (bottom right panel) in operators who worked in COVID-19-free wards and departments (CFO, white) and in frontline COVID-19 operators during the pandemic (FLCO, grey) during the first (I) and the second session (II). *: p<0.05 CFO1 vs FLCO1; °: p<0.05 CFO2 vs FLCO2. ♦: p<0.05 FLCO1 vs FLCO2.


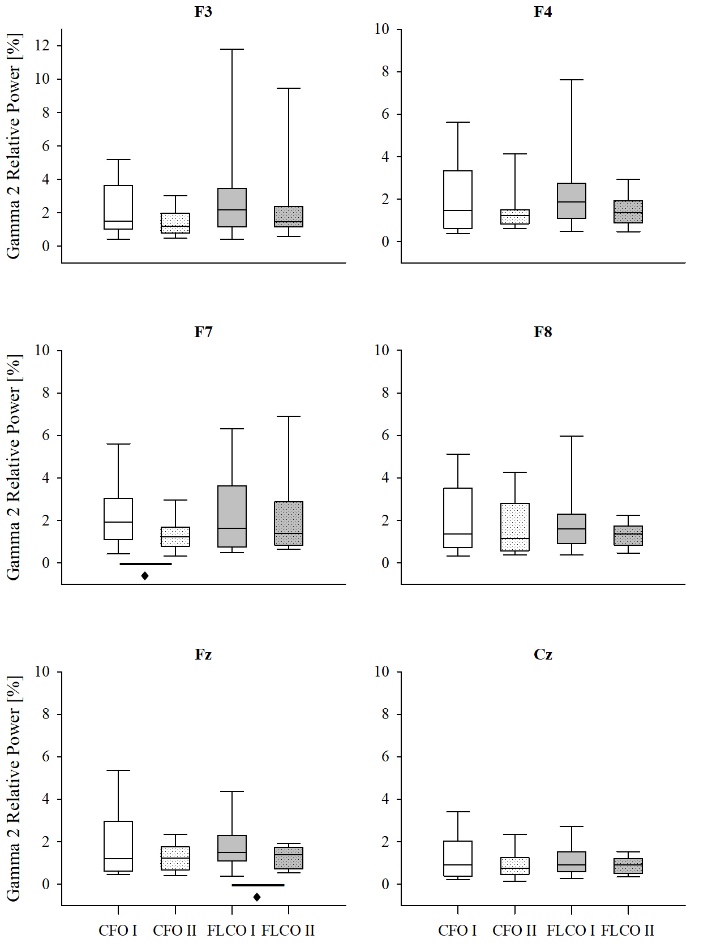

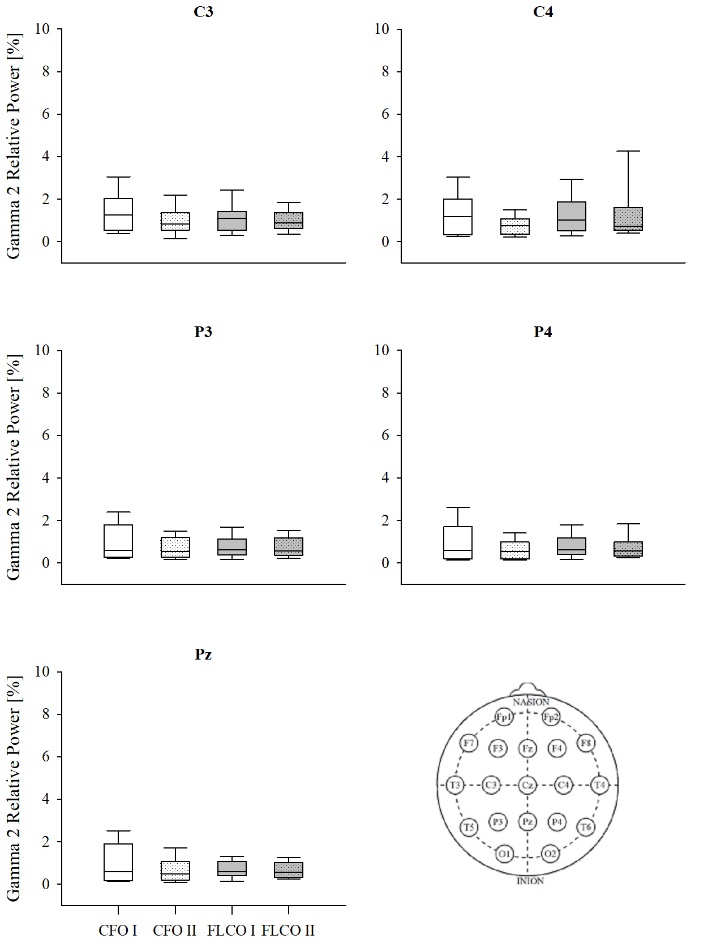

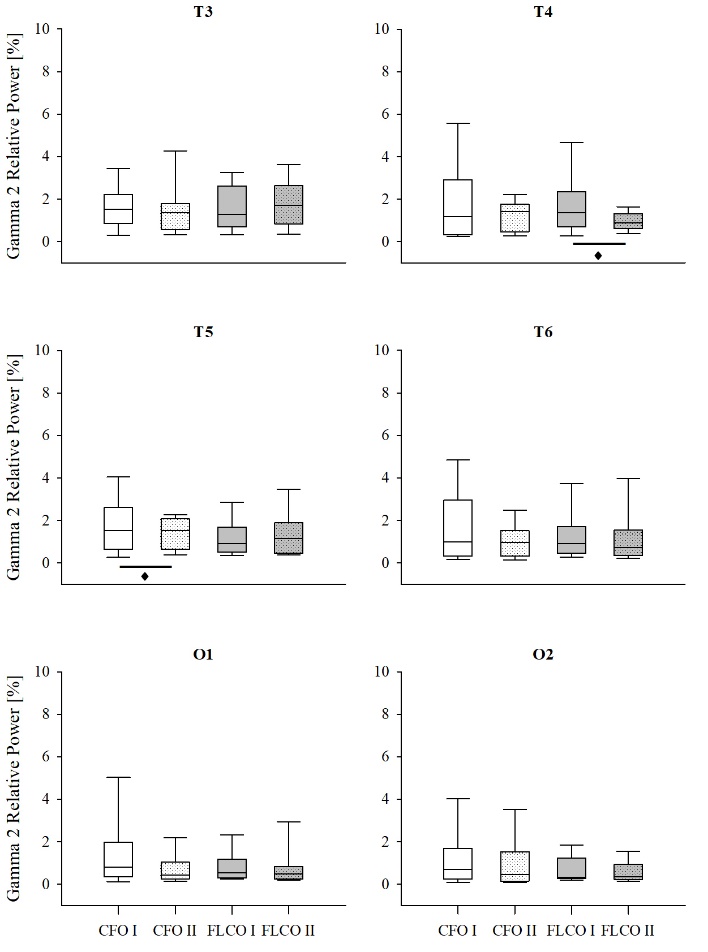


**Figure 9.** Box-and-whisker plot representing the median (line within the box), the interquartile range (length of the box), the 90th and the 10th percentiles (whiskers above and below the box) of the electroencephalographic derived Gamma 2 Relative Power in F3, F4, F7, F8, Fz, C3, C4, Cz, P3, P4, Pz, T3, T4, T5, T6, O1 and O2 electrode in operators who worked in COVID-19-free wards and departments (CFO, white) and in frontline COVID-19 operators during the pandemic (FLCO, grey) during the first (I) and the second session (II). *: p<0.05 CFO1 vs FLCO1; °: p<0.05 CFO2 vs FLCO2; ♦: p<0.05 FLCO1 vs FLCO2.


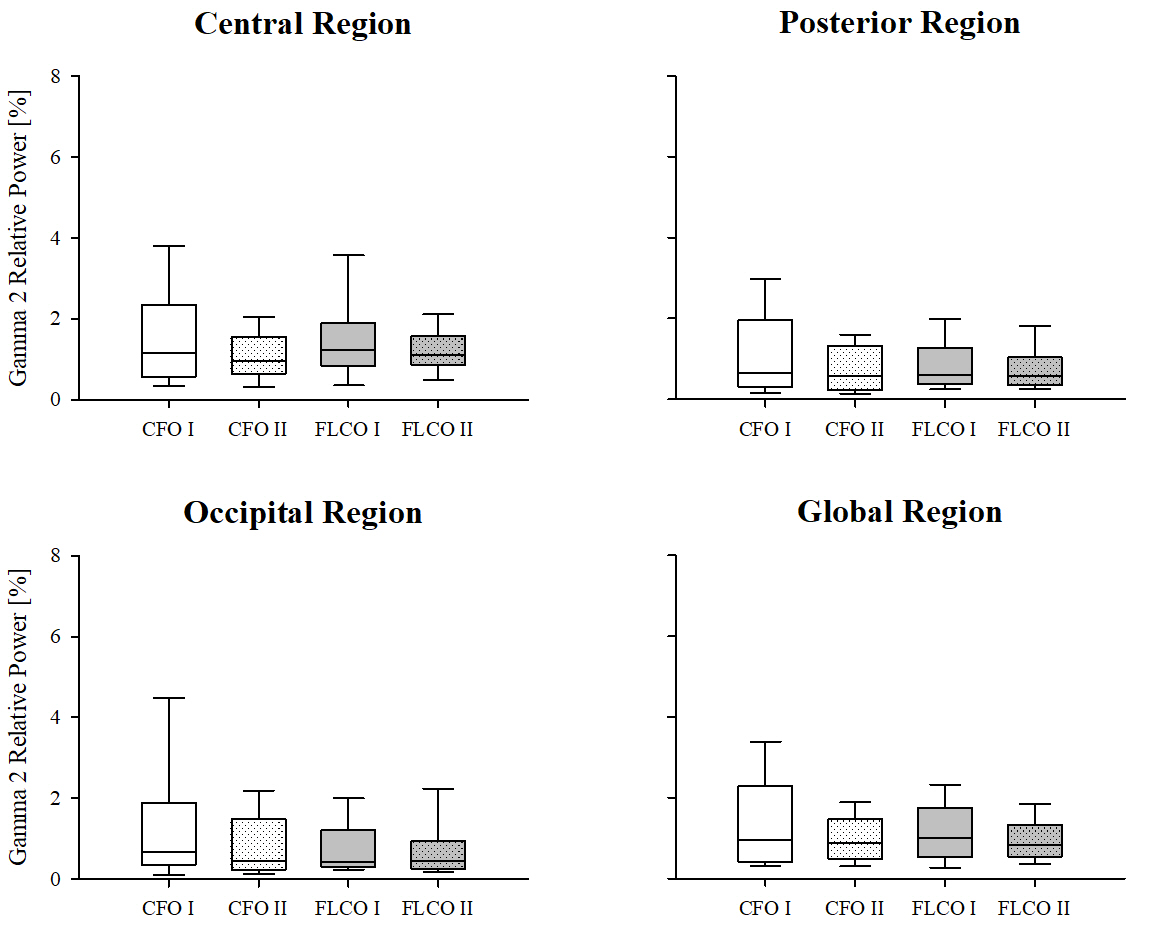


**Figure 10.** Box-and-whisker plot representing the median (line within the box), the interquartile range (length of the box), the 90th and the 10th percentiles (whiskers above and below the box) of the electroencephalographic derived Gamma 2 Relative Power in central (top left panel), posterior (top right panel), occipital (bottom left panel), and global regions (bottom right panel) in operators who worked in COVID-19-free wards and departments (CFO, white) and in frontline COVID-19 operators during the pandemic (FLCO, grey) during the first (I) and the second session (II). *: p<0.05 CFO1 vs FLCO1; °: p<0.05 CFO2 vs FLCO2. ♦: p<0.05 FLCO1 vs FLCO2.


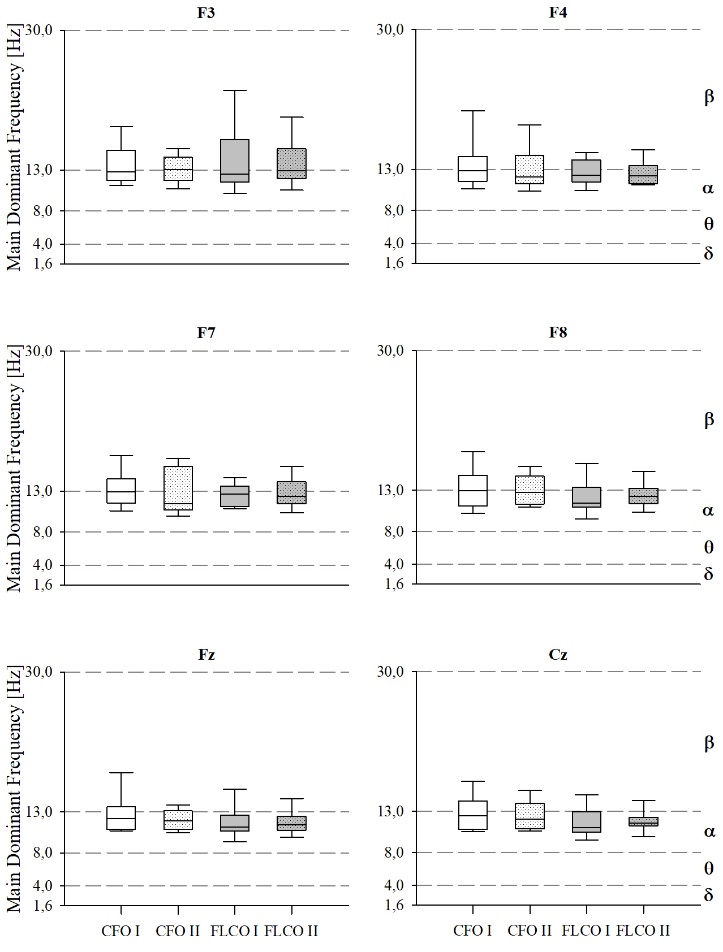

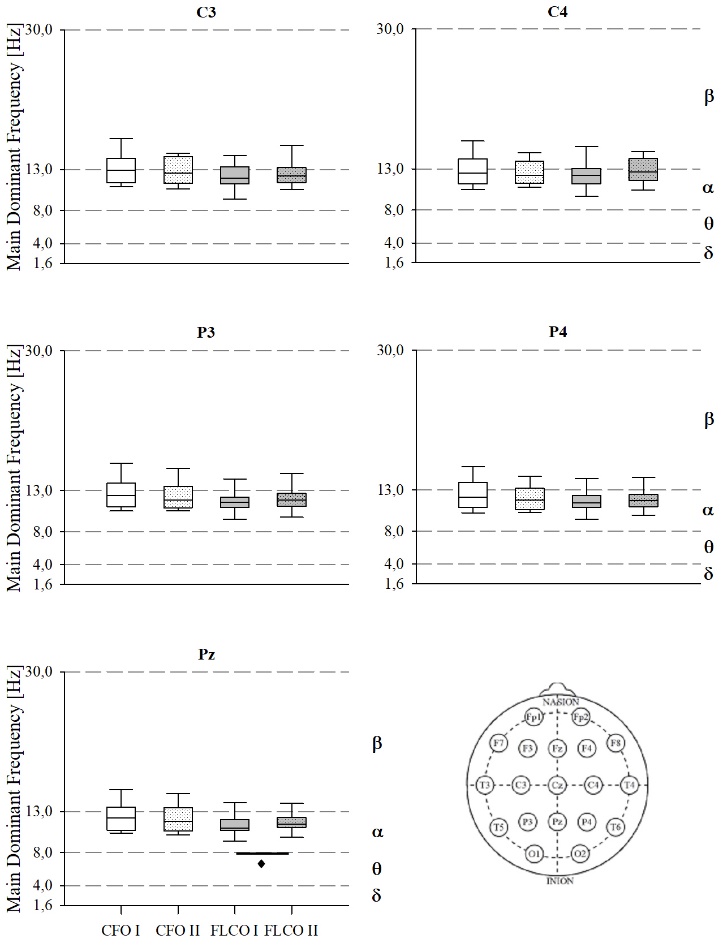

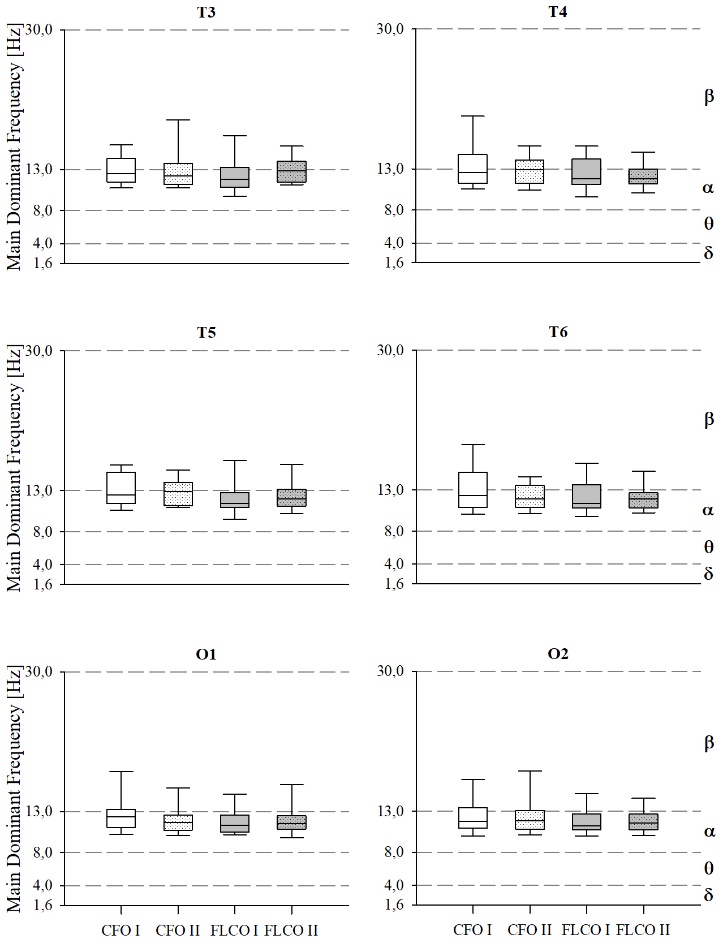


**Figure 11.** Box-and-whisker plot representing the median (line within the box), the interquartile range (length of the box), the 90th and the 10th percentiles (whiskers above and below the box) of the electroencephalographic derived Main Dominant Frequency in F3, F4, F7, F8, Fz, C3, C4, Cz, P3, P4, Pz, T3, T4, T5, T6, O1 and O2 electrode in operators who worked in COVID-19-free wards and departments (CFO, white) and in frontline COVID-19 operators during the pandemic (FLCO, grey) during the first (I) and the second session (II). *: p<0.05 CFO1 vs FLCO1; °: p<0.05 CFO2 vs FLCO2; ♦: p<0.05 FLCO1 vs FLCO2.


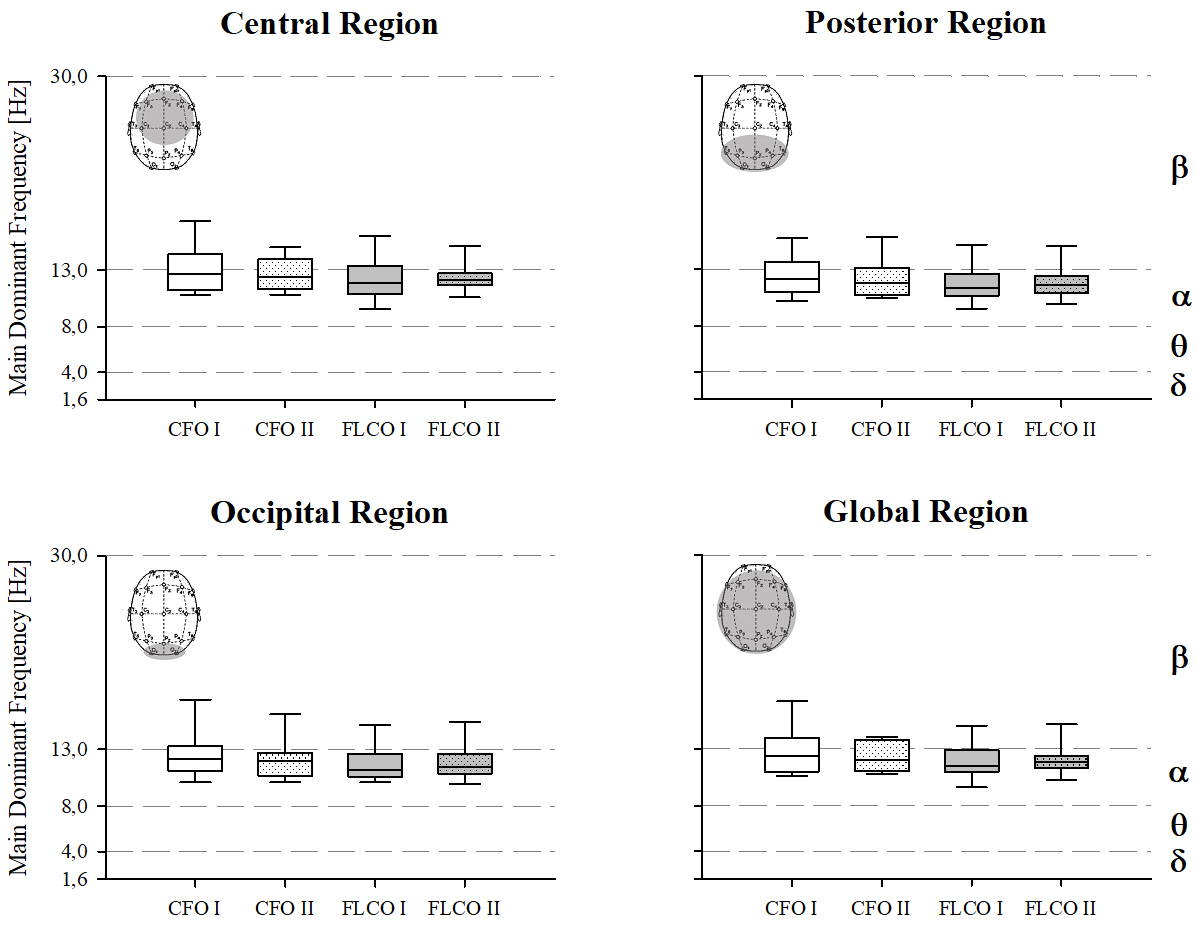


**Figure 12.** Box-and-whisker plot representing the median (line within the box), the interquartile range (length of the box), the 90th and the 10th percentiles (whiskers above and below the box) of the electroencephalographic derived Main Dominant Frequency in central (top left panel), posterior (top right panel), occipital (bottom left panel), and global regions (bottom right panel) in operators who worked in COVID-19-free wards and departments (CFO, white) and in frontline COVID-19 operators during the pandemic (FLCO, grey) during the first (I) and the second session (II). *: p<0.05 CFO1 vs FLCO1; °: p<0.05 CFO2 vs FLCO2. ♦: p<0.05 FLCO1 vs FLCO2.


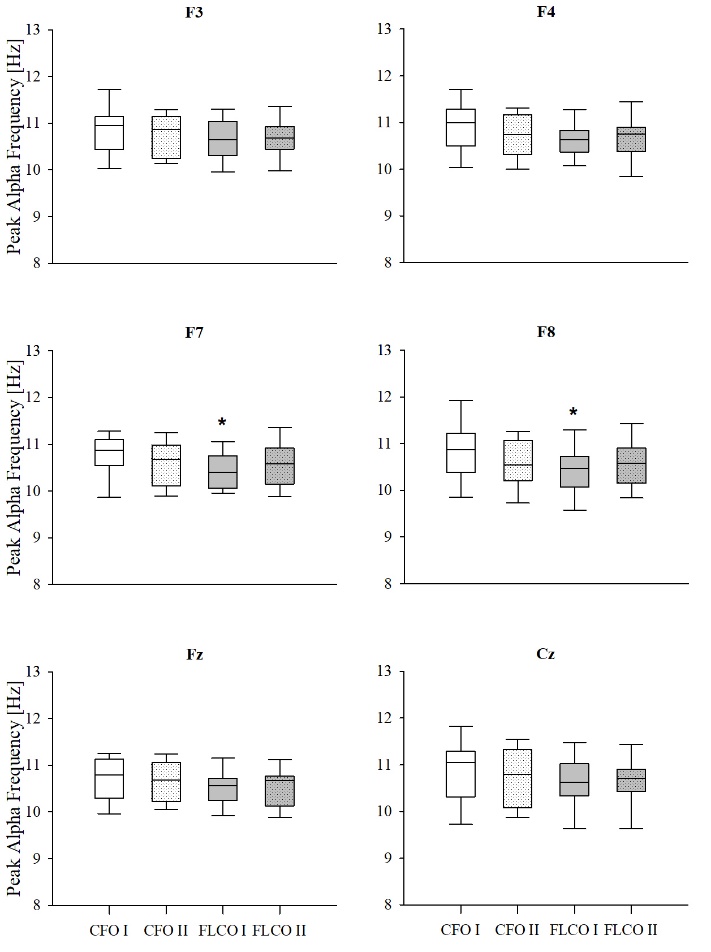

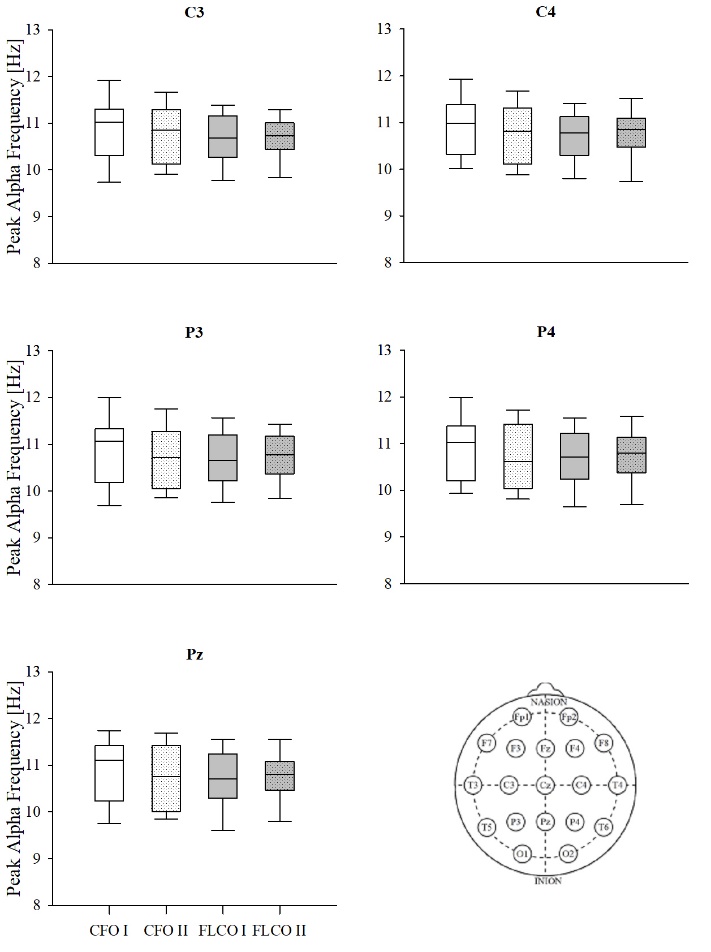

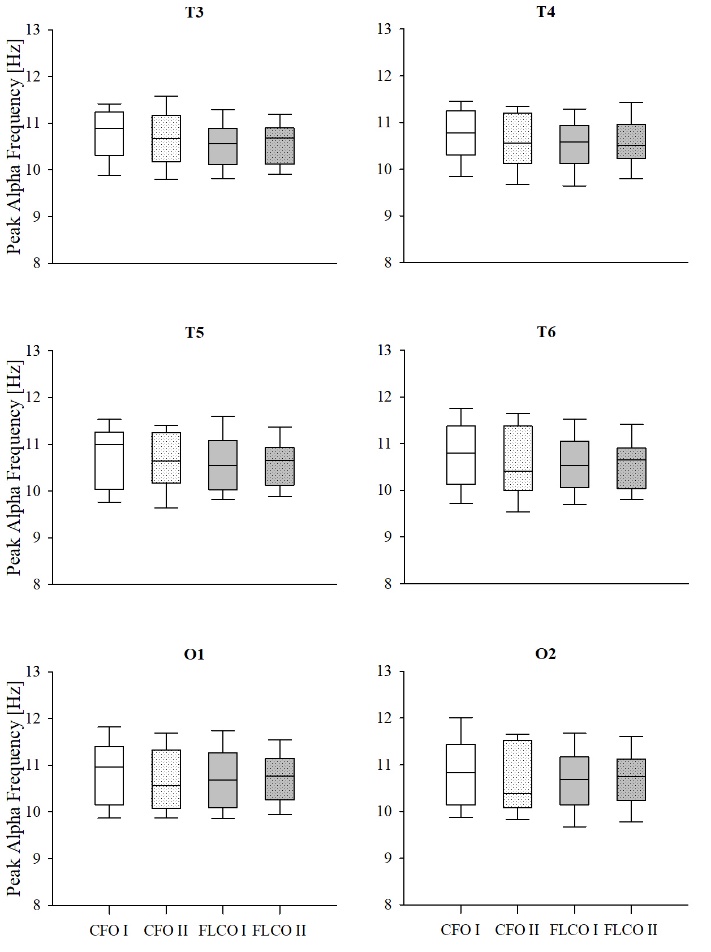


**Figure 13.** Box-and-whisker plot representing the median (line within the box), the interquartile range (length of the box), the 90th and the 10th percentiles (whiskers above and below the box) of the electroencephalographic derived Peak Alpha Frequency in F3, F4, F7, F8, Fz, C3, C4, Cz, P3, P4, Pz, T3, T4, T5, T6, O1 and O2 electrode in operators who worked in COVID-19-free wards and departments (CFO, white) and in frontline COVID-19 operators during the pandemic (FLCO, grey) during the first (I) and the second session (II). *: p<0.05 CFO1 vs FLCO1; °: p<0.05 CFO2 vs FLCO2; ♦: p<0.05 FLCO1 vs FLCO2.


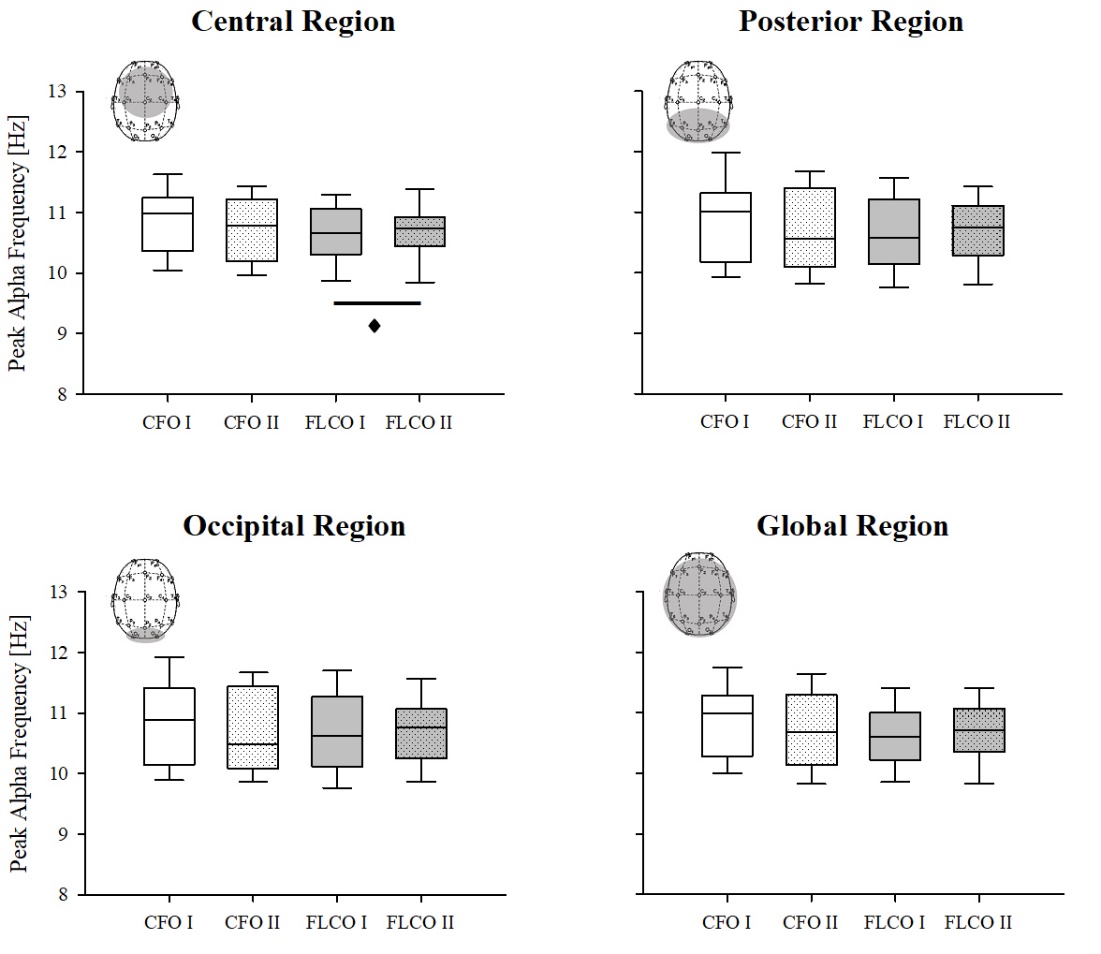


**Figure 14.** Box-and-whisker plot representing the median (line within the box), the interquartile range (length of the box), the 90th and the 10th percentiles (whiskers above and below the box) of the electroencephalographic derived Peak Alpha Frequency in central (top left panel), posterior (top right panel), occipital (bottom left panel), and global regions (bottom right panel) in operators who worked in COVID-19-free wards and departments (CFO, white) and in frontline COVID-19 operators during the pandemic (FLCO, grey) during the first (I) and the second session (II). *: p<0.05 CFO1 vs FLCO1; °: p<0.05 CFO2 vs FLCO2. ♦: p<0.05 FLCO1 vs FLCO2.


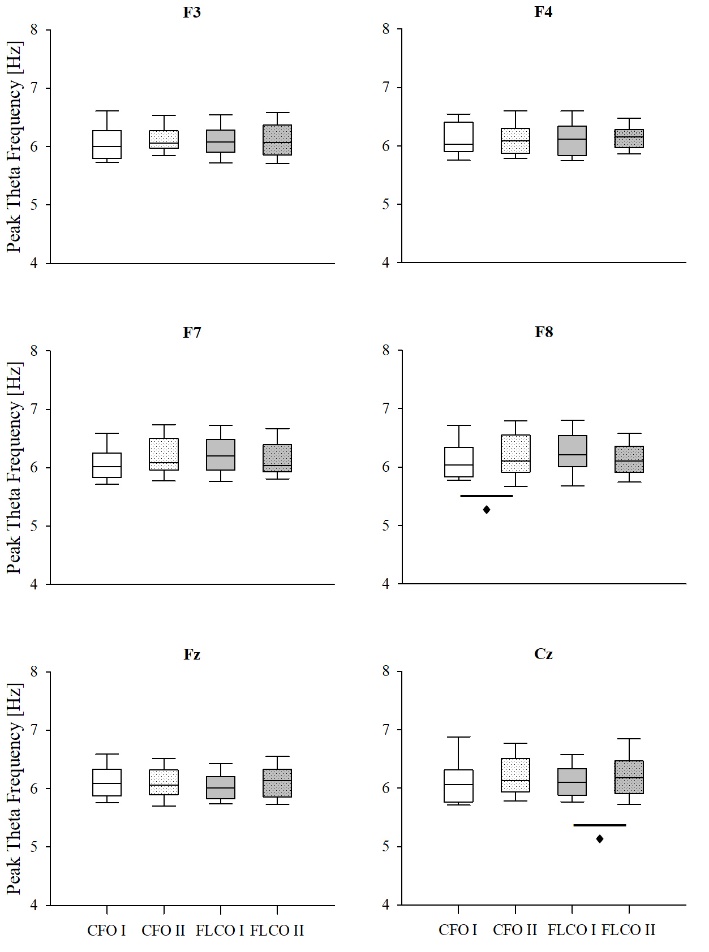

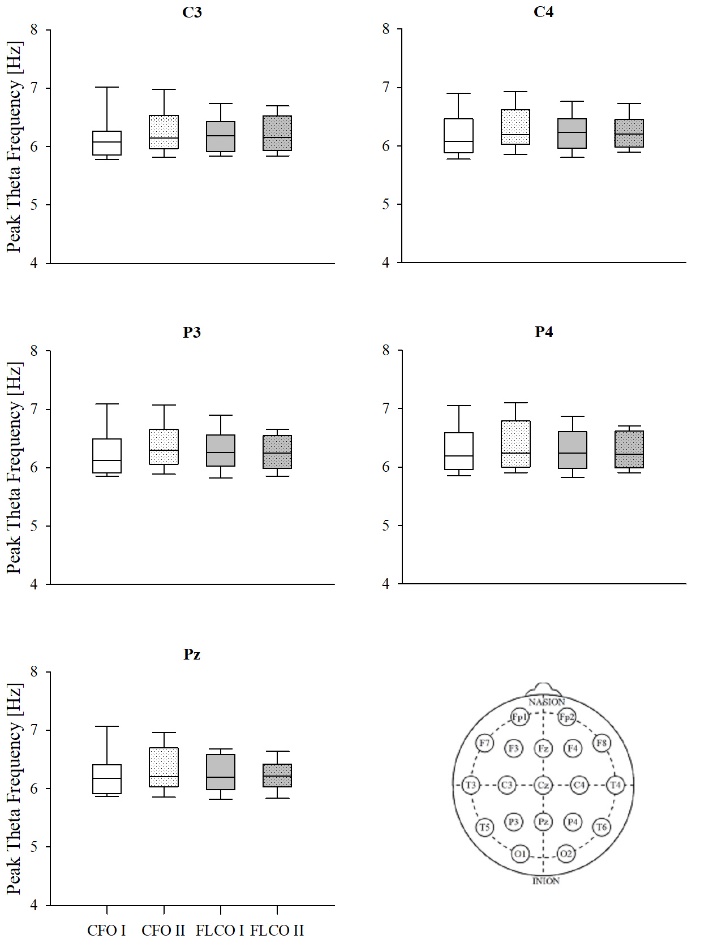

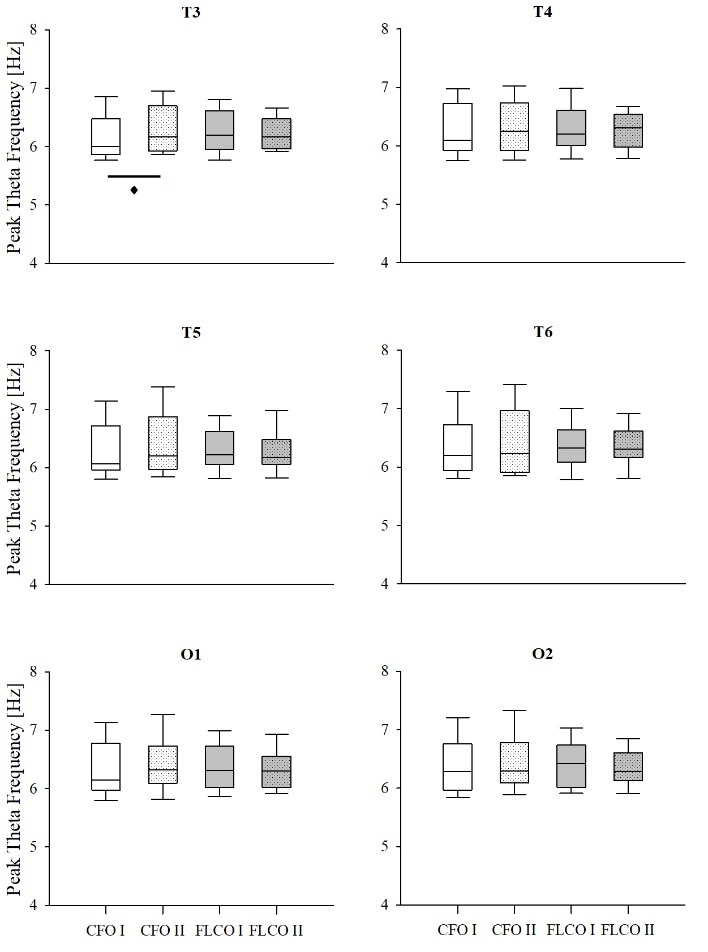


**Figure 15.** Box-and-whisker plot representing the median (line within the box), the interquartile range (length of the box), the 90th and the 10th percentiles (whiskers above and below the box) of the electroencephalographic derived Peak Theta Frequency in F3, F4, F7, F8, Fz, C3, C4, Cz, P3, P4, Pz, T3, T4, T5, T6, O1 and O2 electrode in operators who worked in COVID-19-free wards and departments (CFO, white) and in frontline COVID-19 operators during the pandemic (FLCO, grey) during the first (I) and the second session (II). *: p<0.05 CFO1 vs FLCO1; °: p<0.05 CFO2 vs FLCO2; ♦: p<0.05 FLCO1 vs FLCO2.


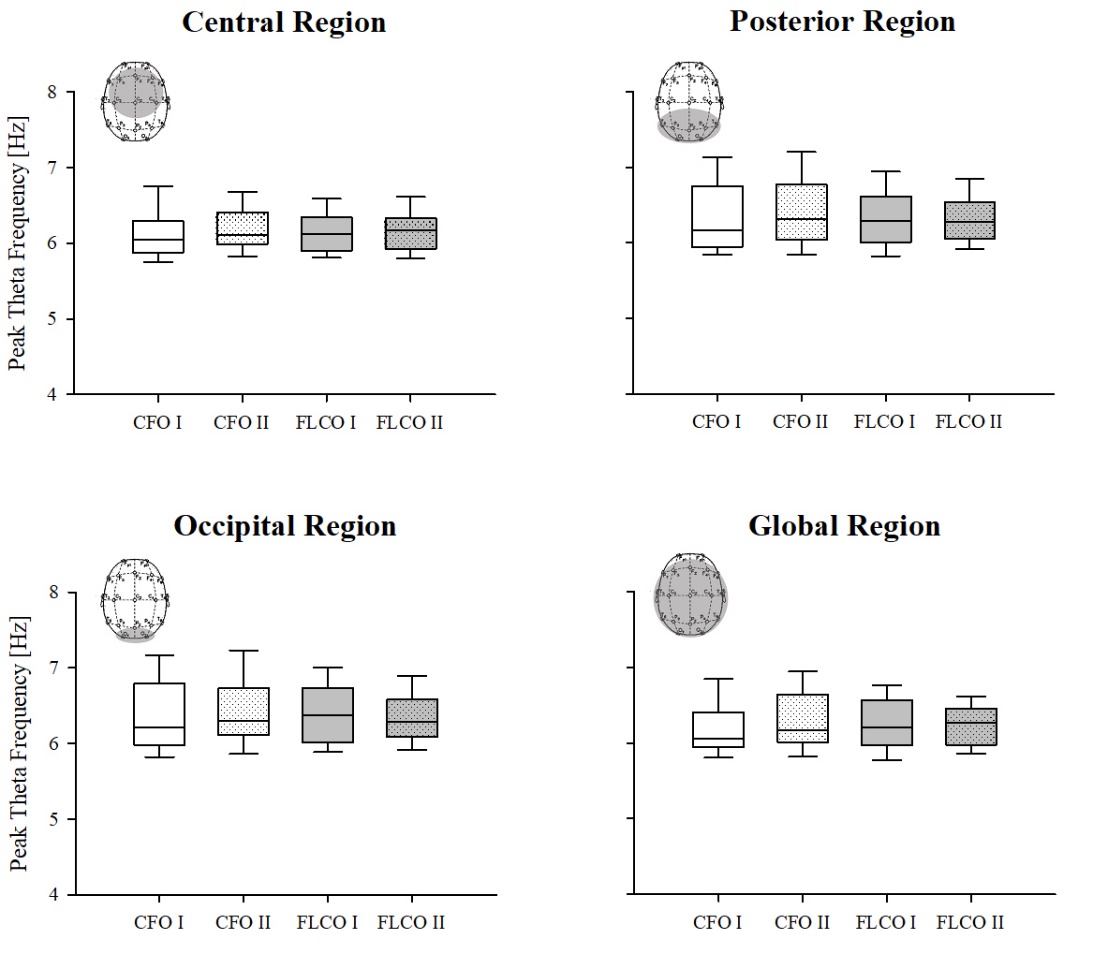


**Figure 16.** Box-and-whisker plot representing the median (line within the box), the interquartile range (length of the box), the 90th and the 10th percentiles (whiskers above and below the box) of the electroencephalographic derived Peak Theta Frequency in central (top left panel), posterior (top right panel), occipital (bottom left panel), and global regions (bottom right panel) in operators who worked in COVID-19-free wards and departments (CFO, white) and in frontline COVID-19 operators during the pandemic (FLCO, grey) during the first (I) and the second session (II). *: p<0.05 CFO1 vs FLCO1; °: p<0.05 CFO2 vs FLCO2. ♦: p<0.05 FLCO1 vs FLCO2.


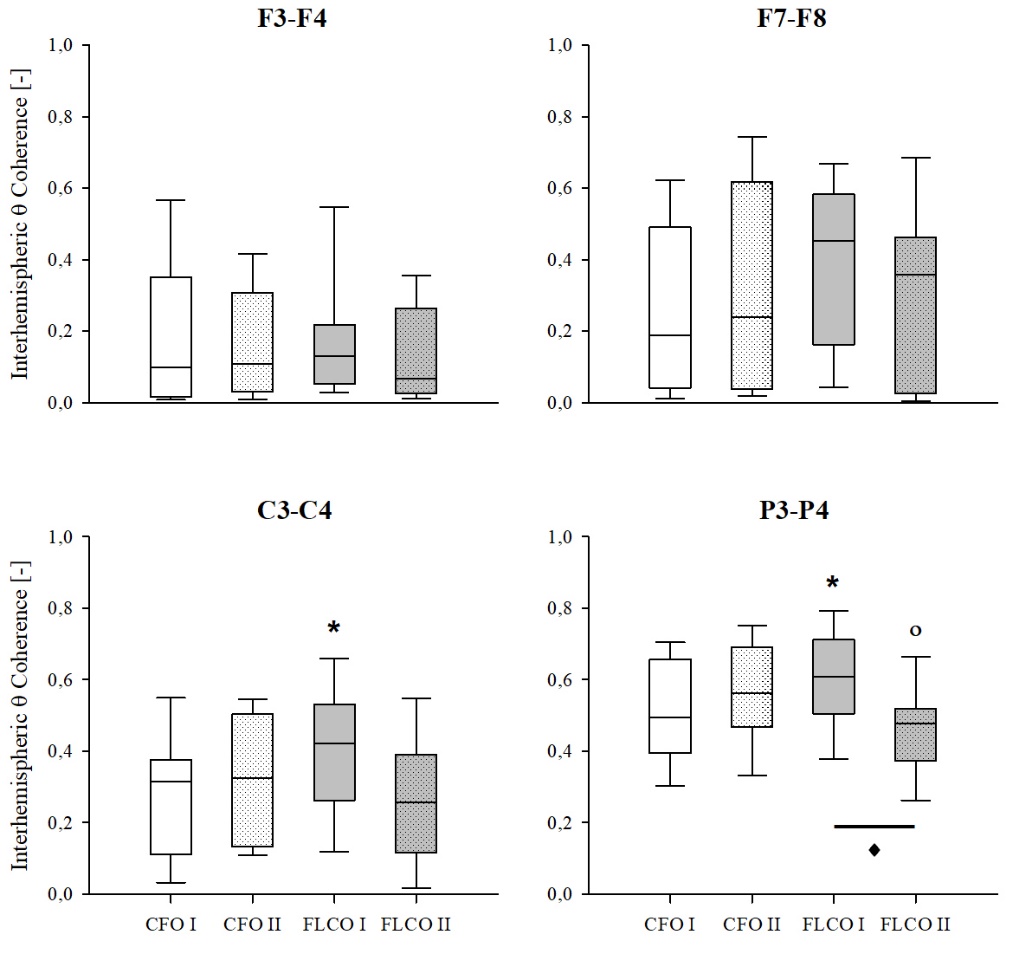

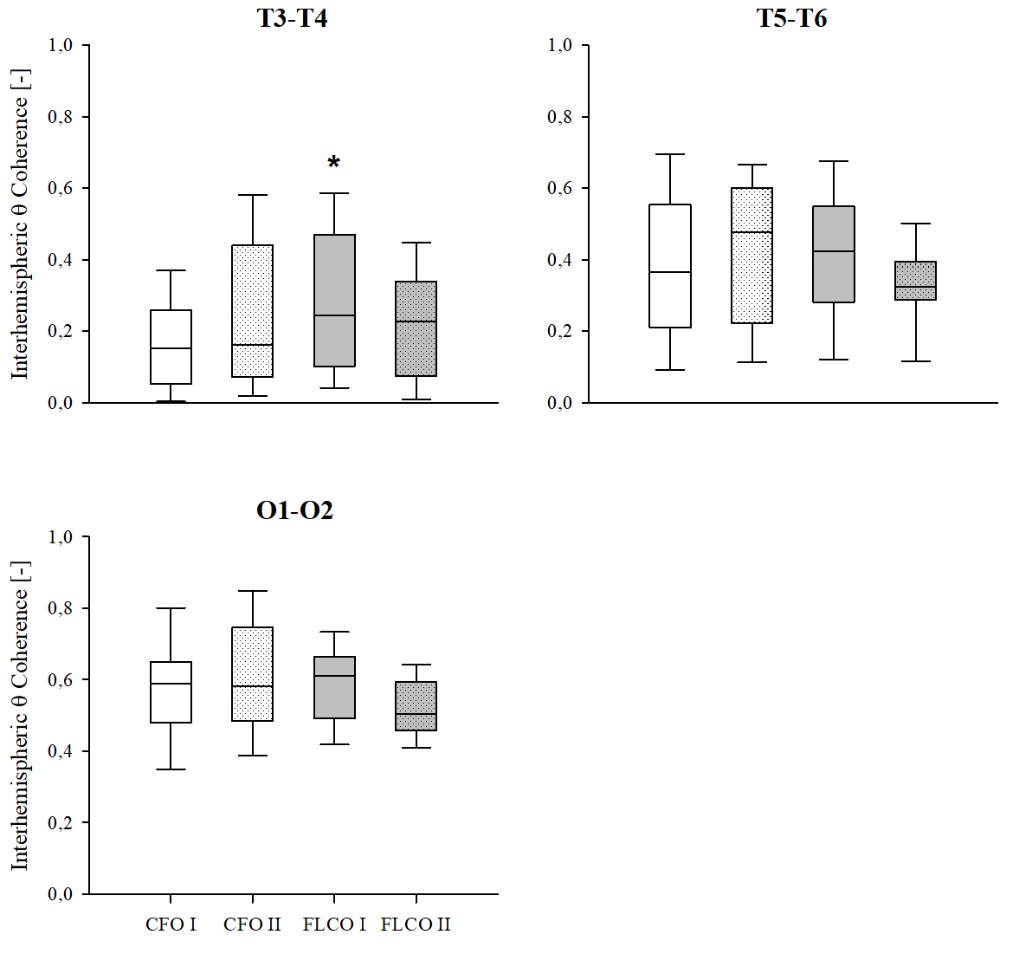


**Figure 17.** Box-and-whisker plot representing the median (line within the box), the interquartile range (length of the box), the 90th and the 10th percentiles (whiskers above and below the box) of the electroencephalographic derived interhemispheric theta coherence in F3-F4, F7-F8, C3-C4, P3-P4, T4-T5, T5-T6 and O1-O2 electrodes in operators who worked in COVID-19-free wards and departments (CFO, white) and in frontline COVID-19 operators during the pandemic (FLCO, grey) during the first (I) and the second session (II). *: p<0.05 CFO1 vs FLCO1; °: p<0.05 CFO2 vs FLCO2; ♦: p<0.05 FLCO1 vs FLCO2.


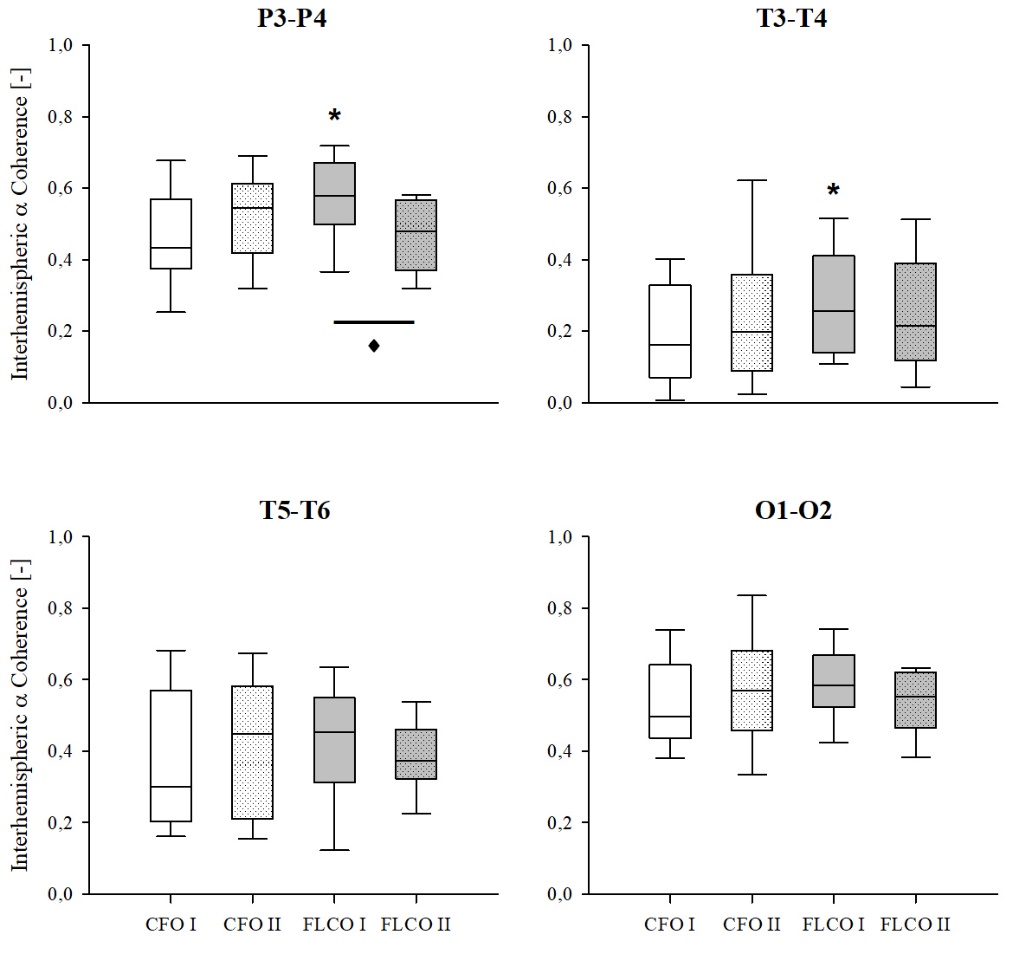


**Figure 18.** Box-and-whisker plot representing the median (line within the box), the interquartile range (length of the box), the 90th and the 10th percentiles (whiskers above and below the box) of the electroencephalographic derived interhemispheric alpha coherence in P3-P4, T3-T4, T5-T6 and O1-O2 electrodes in operators who worked in COVID-19-free wards and departments (CFO, white) and in frontline COVID-19 operators during the pandemic (FLCO, grey) during the first (I) and the second session (II). *: p<0.05 CFO1 vs FLCO1; °: p<0.05 CFO2 vs FLCO2; ♦: p<0.05 FLCO1 vs FLCO2.


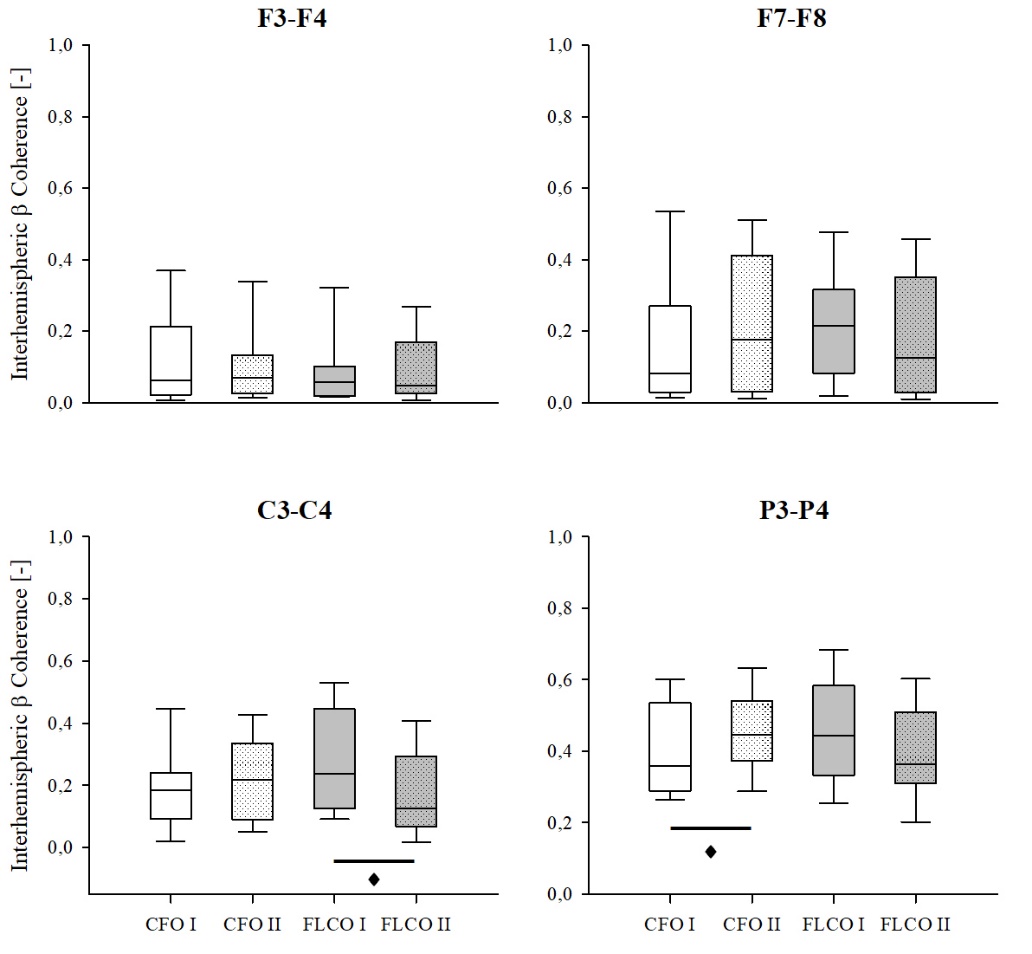

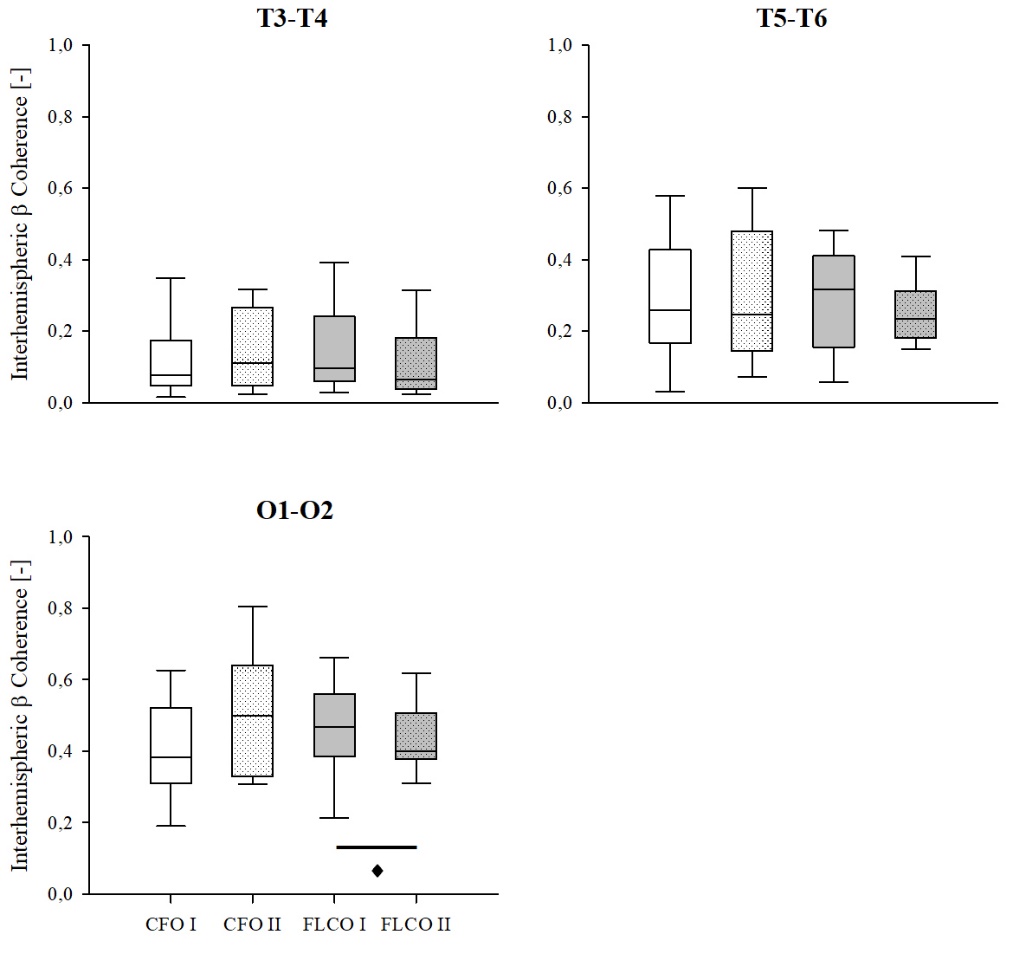


**Figure 19.** Box-and-whisker plot representing the median (line within the box), the interquartile range (length of the box), the 90th and the 10th percentiles (whiskers above and below the box) of the electroencephalographic derived interhemispheric beta coherence in F3-F4, F7-F8, C3-C4, P3-P4, T4-T5, T5-T6 and O1-O2 electrodes in operators who worked in COVID-19-free wards and departments (CFO, white) and in frontline COVID-19 operators during the pandemic (FLCO, grey) during the first (I) and the second session (II). *: p<0.05 CFO1 vs FLCO1; °: p<0.05 CFO2 vs FLCO2; ♦: p<0.05 FLCO1 vs FLCO2.


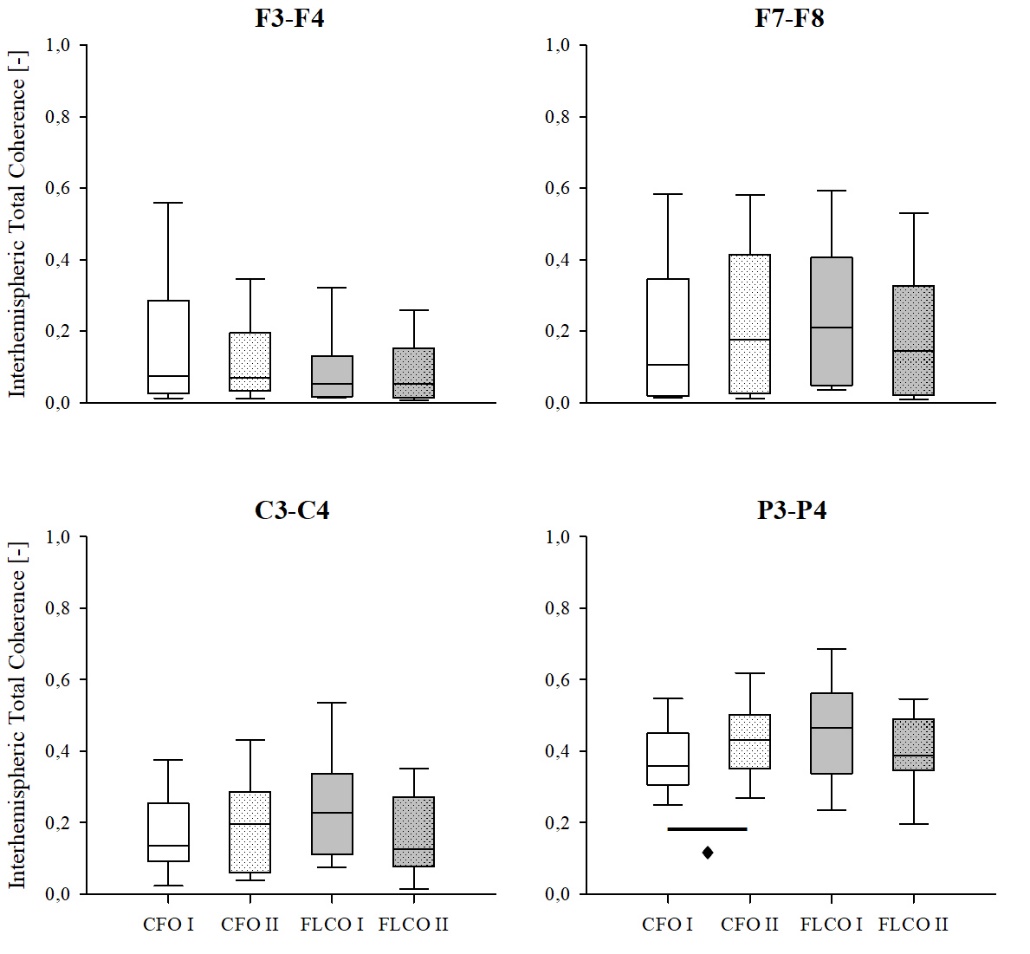

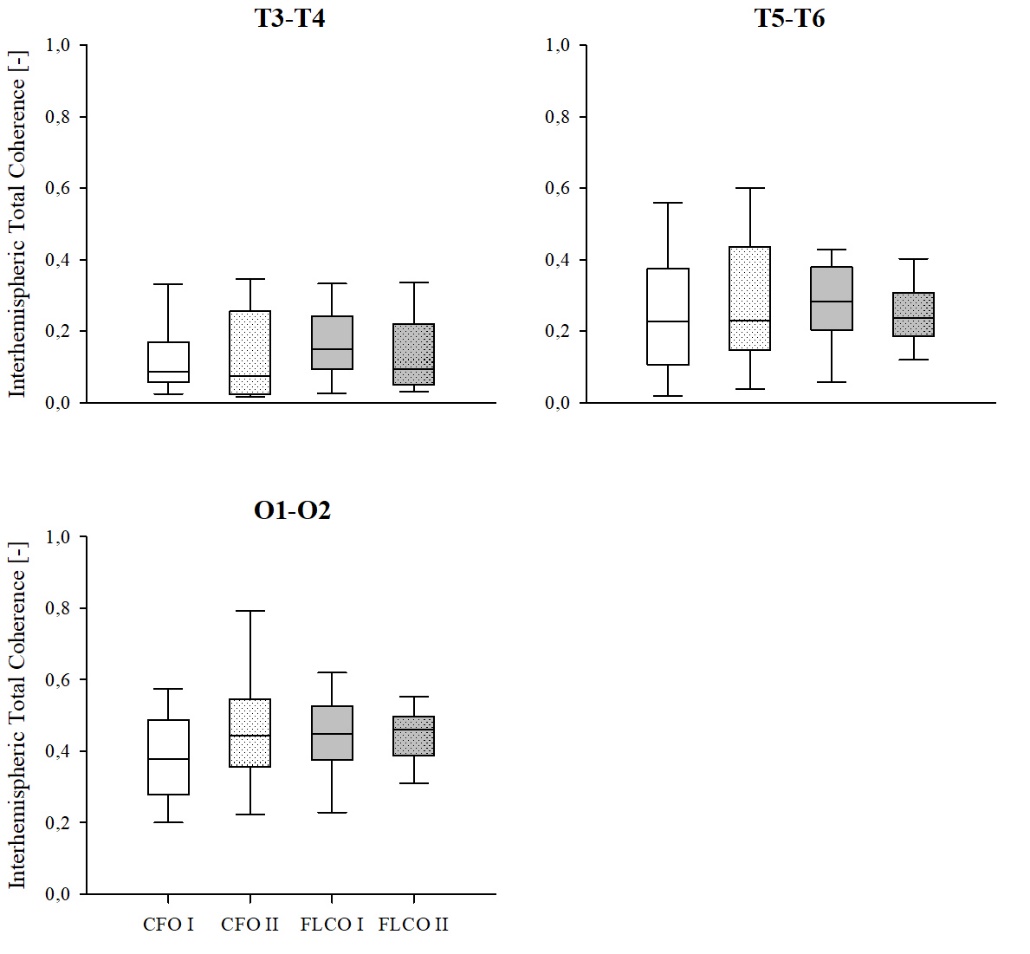


**Figure 20.** Box-and-whisker plot representing the median (line within the box), the interquartile range (length of the box), the 90th and the 10th percentiles (whiskers above and below the box) of the electroencephalographic derived interhemispheric total coherence in F3-F4, F7-F8, C3-C4, P3-P4, T4-T5, T5-T6 and O1-O2 electrodes in operators who worked in COVID-19-free wards and departments (CFO, white) and in frontline COVID-19 operators during the pandemic (FLCO, grey) during the first (I) and the second session (II). *: p<0.05 CFO1 vs FLCO1; °: p<0.05 CFO2 vs FLCO2; ♦: p<0.05 FLCO1 vs FLCO2.


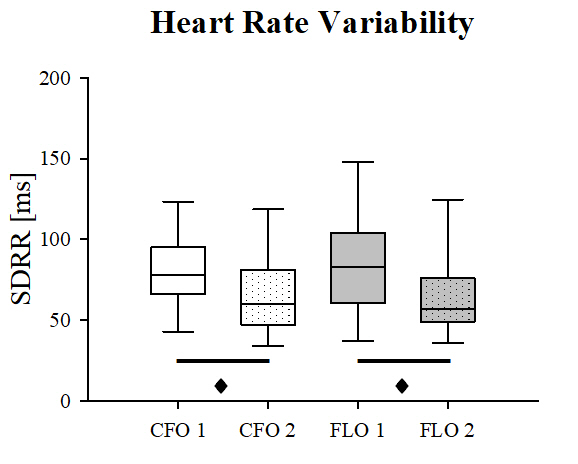


**Figure 21.** Box-and-whisker plot representing the median (line within the box), the interquartile range (length of the box), the 90th and the 10th percentiles (whiskers above and below the box) of the electroencephalographic derived Heart Rate Variability (Standard Deviation of R-R Intervals) in operators who worked in COVID-19-free wards and departments (CFO, white) and in frontline COVID-19 operators during the pandemic (FLCO, grey) during the first (I) and the second session (II). *: p<0.05 CFO1 vs FLCO1; °: p<0.05 CFO2 vs FLCO2; ♦: p<0.05 FLCO1 vs FLCO2.


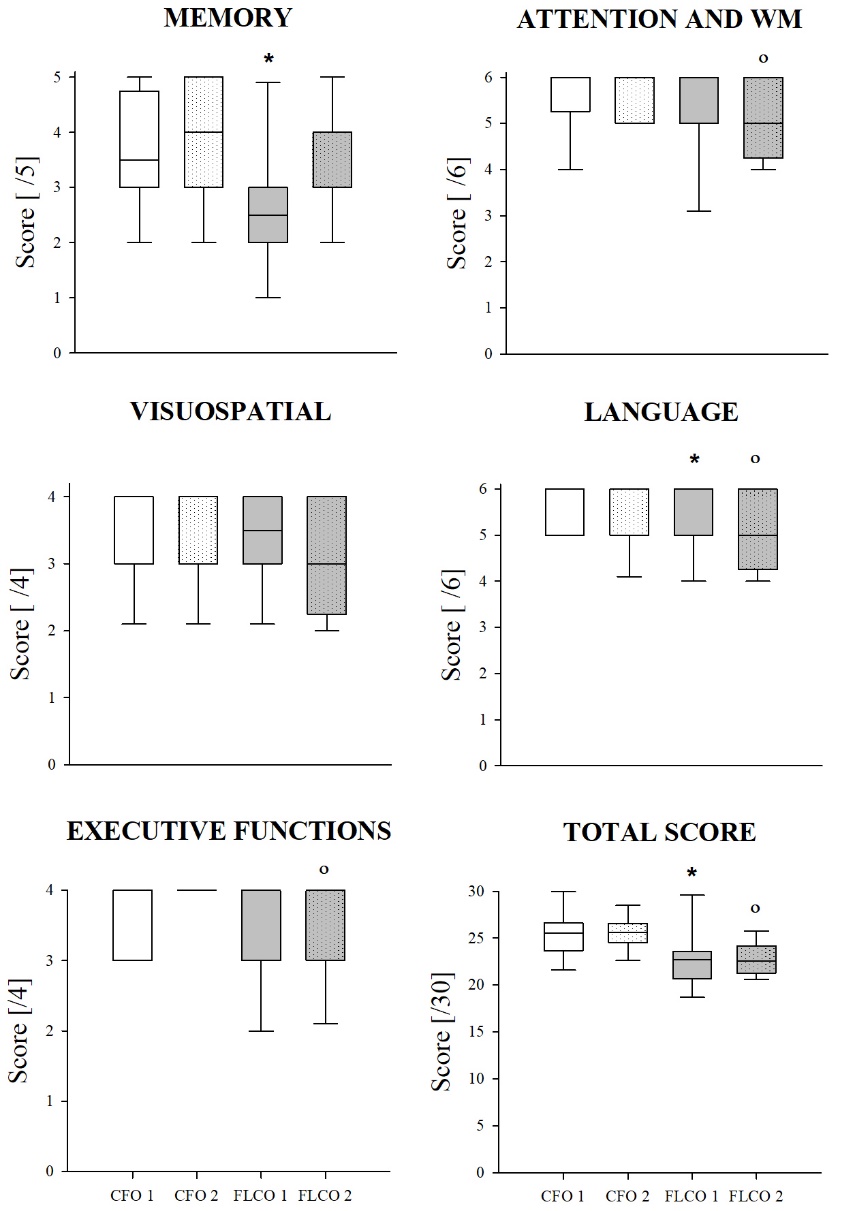


**Figure 22.** Box-and-whisker plot representing the median (line within the box), the interquartile range (length of the box), the 90th and the 10th percentiles (whiskers above and below the box) of Montreal Cognitive Assessment Test Memory, Visuospatial Ability, Executive Functions, Attention and Working Memory, Language and Total Scores in operators who worked in COVID-19-free wards and departments (CFO, white) and in frontline COVID-19 operators during the pandemic (FLCO, grey) during the first (1) and the second session (2). *: p<0.05 CFO1 vs FLCO1; °: p<0.05 CFO2 vs FLCO2; ♦: p<0.05 FLCO1 vs FLCO2.


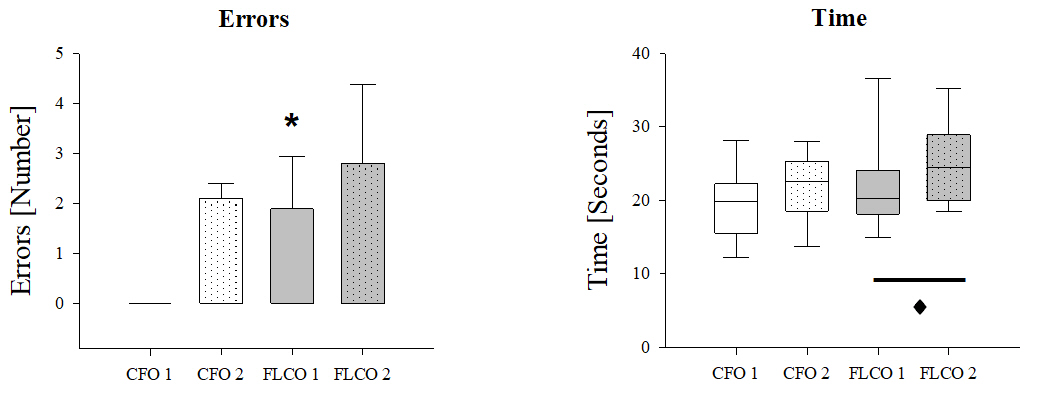


**Figure 23.** Box-and-whisker plot representing the median (line within the box), the interquartile range (length of the box), the 90th and the 10th percentiles (whiskers above and below the box) of Stroop Color and Word Test number of errors and time duration in operators who worked in COVID-19-free wards and departments (CFO, white) and in frontline COVID-19 operators during the pandemic (FLCO, grey) during the first (1) and the second session (2). *: p<0.05 CFO1 vs FLCO1; °: p<0.05 CFO2 vs FLCO2; ♦: p<0.05 FLCO1 vs FLCO2.


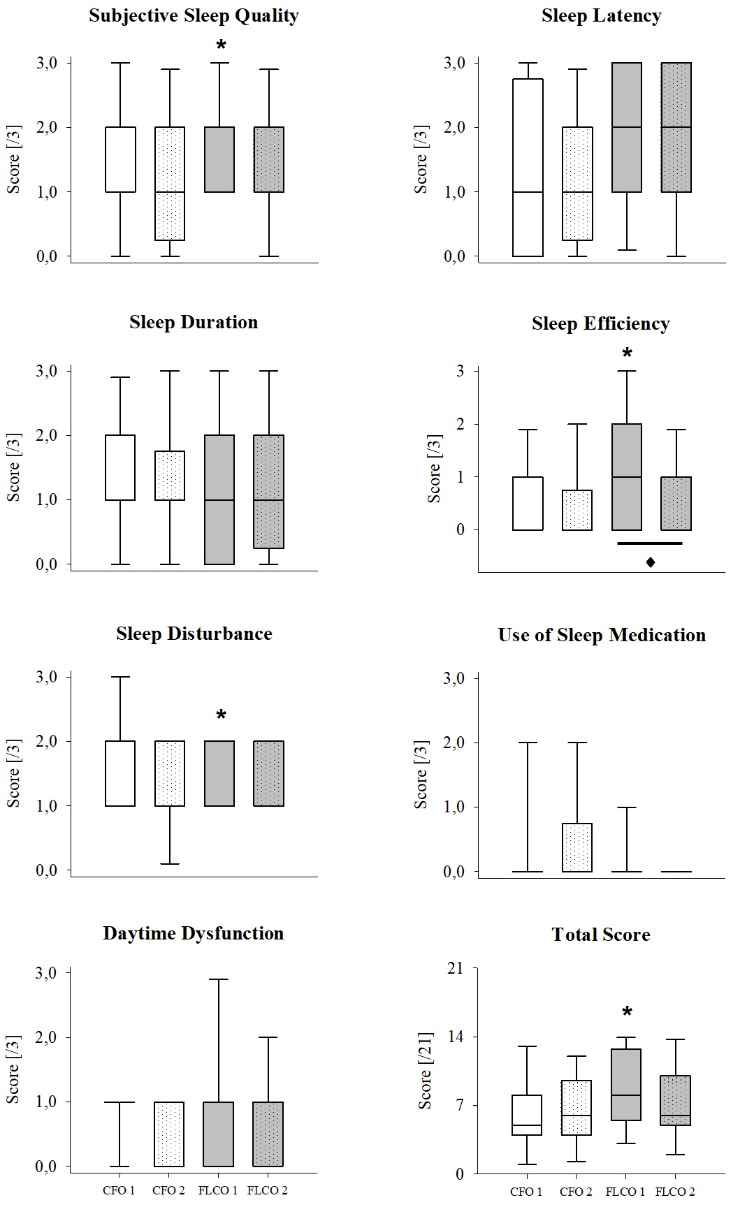


**Figure 24.** Box-and-whisker plot representing the median (line within the box), the interquartile range (length of the box), the 90th and the 10th percentiles (whiskers above and below the box) of Pittsburgh Sleep Quality Index subjective sleep quality, sleep latency, sleep duration, sleep efficiency, sleep disturbance, use of sleep medication, daytime dysfunction and total scores in operators who worked in COVID-19-free wards and departments (CFO, white) and in frontline COVID-19 operators during the pandemic (FLCO, grey) during the first (1) and the second session (2). *: p<0.05 CFO1 vs FLCO1; °: p<0.05 CFO2 vs FLCO2; ♦: p<0.05 FLCO1 vs FLCO2.


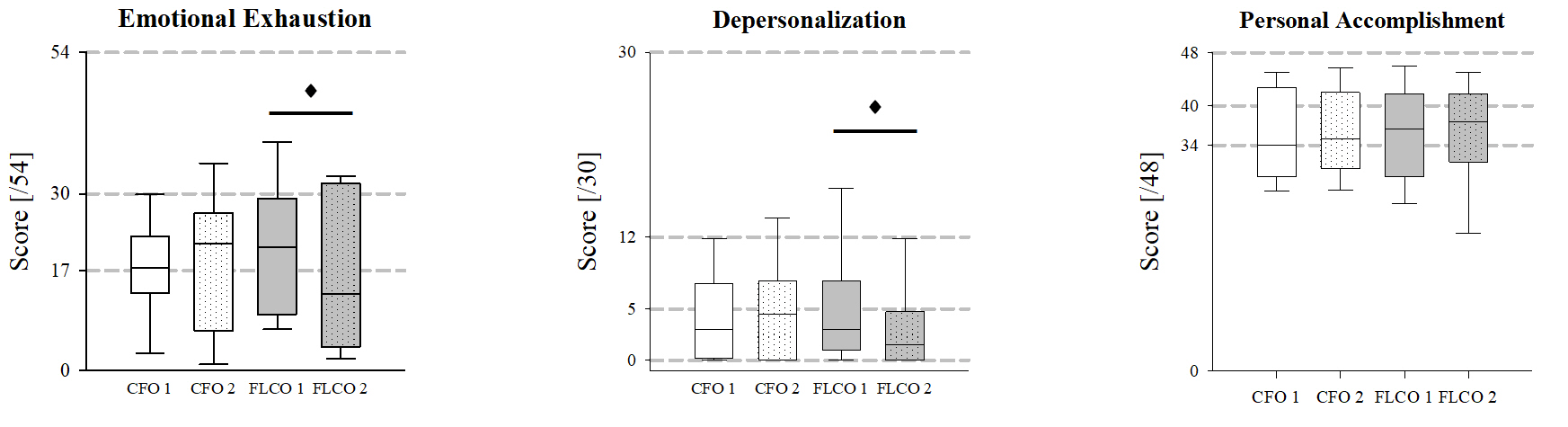


**Figure 25.** Box-and-whisker plot representing the median (line within the box), the interquartile range (length of the box), the 90th and the 10th percentiles (whiskers above and below the box) of MBI-GS Emotional Exhaustion, Depersonalization and Personal Accomplishment scores in operators who worked in COVID-19-free wards and departments (CFO, white) and in frontline COVID-19 operators during the pandemic (FLCO, grey) during the first (1) and the second session (2). *: p<0.05 CFO1 vs FLCO1; °: p<0.05 CFO2 vs FLCO2; ♦: p<0.05 FLCO1 vs FLCO2.
